# Supplementary material for: Effects of Chronic Inflammatory Activation of Murine and Human Arterial Endothelial Cells at Normal Lipoprotein and Cholesterol Levels In Vivo and In Vitro
Source: Cells. 2024 Apr 30;13(9):773. doi: 10.3390/cells13090773 (PMC11083315; doi:10.3390/cells13090773)
Supplement: Supplementary file 1 [file cells-13-00773-s001.zip › cells-2814350-supplementary.pdf]

Effects of chronic inflammatory activation of murine and human arterial endothelial cells at normal lipoprotein and cholesterol levels in vivo and in vitro

Marion Mussbacher, José Basílio, Barbora Belakova, Anita Pirabe, Elisabeth Ableitner, Manuel Campos-Medina and Johannes A. Schmid

Supplementary Methods 1

Supplementary Figure S1. Confirmatory data on the mouse model of arterial endothelial inflammation 3

Supplementary Figure S2. Confirmation of the transgene expression in the endothelial layer after inducing the Cre recombinase activity by tamoxifen injection into mice. 4

Supplementary Figure S3. Deconvolution of cell types from aorta samples using CIBERSORTx ... 5

Supplementary Figure S4. Network analysis of differentially regulated genes after inflammatory activation of endothelial cells, which have roles in Coronavirus infection pathways. 6

Supplementary Figure S5: ACE2 expression in HUVECs upon inflammatory activation 6

Suppl. Figure S6: Apoptosis in HUVECs 7

Suppl. Figure S7: Transcriptomic changes of HUVECs after short (5h) and long-term (9d) treatment with TNFα 8

Suppl. Figure S8: Pathway analysis of differentially expressed genes in isolated human endothelial cells after prolonged treatment with TNFα: 9

Suppl. Figure S9: Re-analysis of the dataset GSE77962 for ACE2 expression 10

Suppl. Table S1: Comparison of the effects of endothelial caIKK2 expression in murine aortas of ApoE wild-type or knock-out background 11

Suppl. Table S2: All biological functions altered in murine aortas after inflammatory activation of endothelial cells on ApoE-wild-type background as assessed by Ingenuity Pathway Analysis 13

Supplementary Methods

Immuno-staining of murine aortas for transgene expression

Cre-negative and BMX-CreERT2-positive mice harboring the floxed-stop-caIKK2-IRES-GFP transgene were injected on 5 consecutive days with tamoxifen as described in the main Methods section. Aortas were isolated by microscope-assisted dissection, perfusion-fixed with 4% paraformaldehyde, followed by dehydration and paraffination according to standard procedure. Paraffin-embedded tissue was cut

by a microtome to 4 µm thick sections and placed on cover slips. Sections were heated in a dry oven at 90°C for 10 min, followed by standard de-paraffination and rehydration.
Antigen retrieval war performed in a boiling solution of 2mM Citric acid, 9mM Trisodium Citrate (pH 6.0), and 0.01% v/v Tween20 buffer for 10 min, and incubated in the same buffer for 30 min without the heat source. After the incubation time, the slides were rinsed using cold tap water.
For the immunostaining of the GFP, which is expressed from the transgene downstream of an internal ribosomal entry site (IRES), the areas enclosing the aortae were circled with a hydrophobic pen (DAIDO SANGYO, Tokyo, Japan). The sections were washed twice, for 5 min each time, with TBS + 0.025% v/v Tween 20 and each section was incubated with a blocking buffer consisting of TBS + 0.025% v/v Tween20 + 3% BSA for 60 mins. After shaking off the blocking solution, the anti-GFP antibody solution (TBS + 0.025% v/v Tween20 + 3% BSA) was applied. The GFP antibody (rabbit IgG, Santa Cruz Biotechnology, Dallas, U.S.A., SC-8334) was used according to the manufacturer guidelines, which suggested a 1:250 dilution and incubation overnight at 4°C. Slides were washed three times for 10 min with TBS + 0.025% v/v Tween 20. Thereafter, the sections were incubated for 60 min at room temperature with a secondary antibody solution consisting of a TBS + 0.025% v/v Tween20 + 3% BSA buffer. The secondary antibody, linked to a Dylight 650 fluorophore, was issued in a 1:250 dilution, according to the manufacturer recommendations (Thermo Fisher Goat-anti-rabbit IgG #84546). The slides were washed twice in TBS, 10 min each time. The slides were mounted using a 125 g/L glycerin solution. Finally, the edges of the slide were sealed with nail polish.
Imaging was done with an Olympus BX61VS slide scanner (Tokyo, Japan), equipped with an Olympus XM10 camera, an Olympus BX – UCB motorized controller, and an XCT10A light 200W Mercury lamp (Massachusetts, U.S.A., Lumen Dynamics Group Inc.). The setup was controlled by the VS-ASW-S6 2.6 / VS-FLUO 2.9 Olympus software. Two images were taken for each aorta section, one image using the FITC configuration (capturing the autofluorescence of the internal elastic lamina), and the remaining image was taken using the Cy5 configuration.

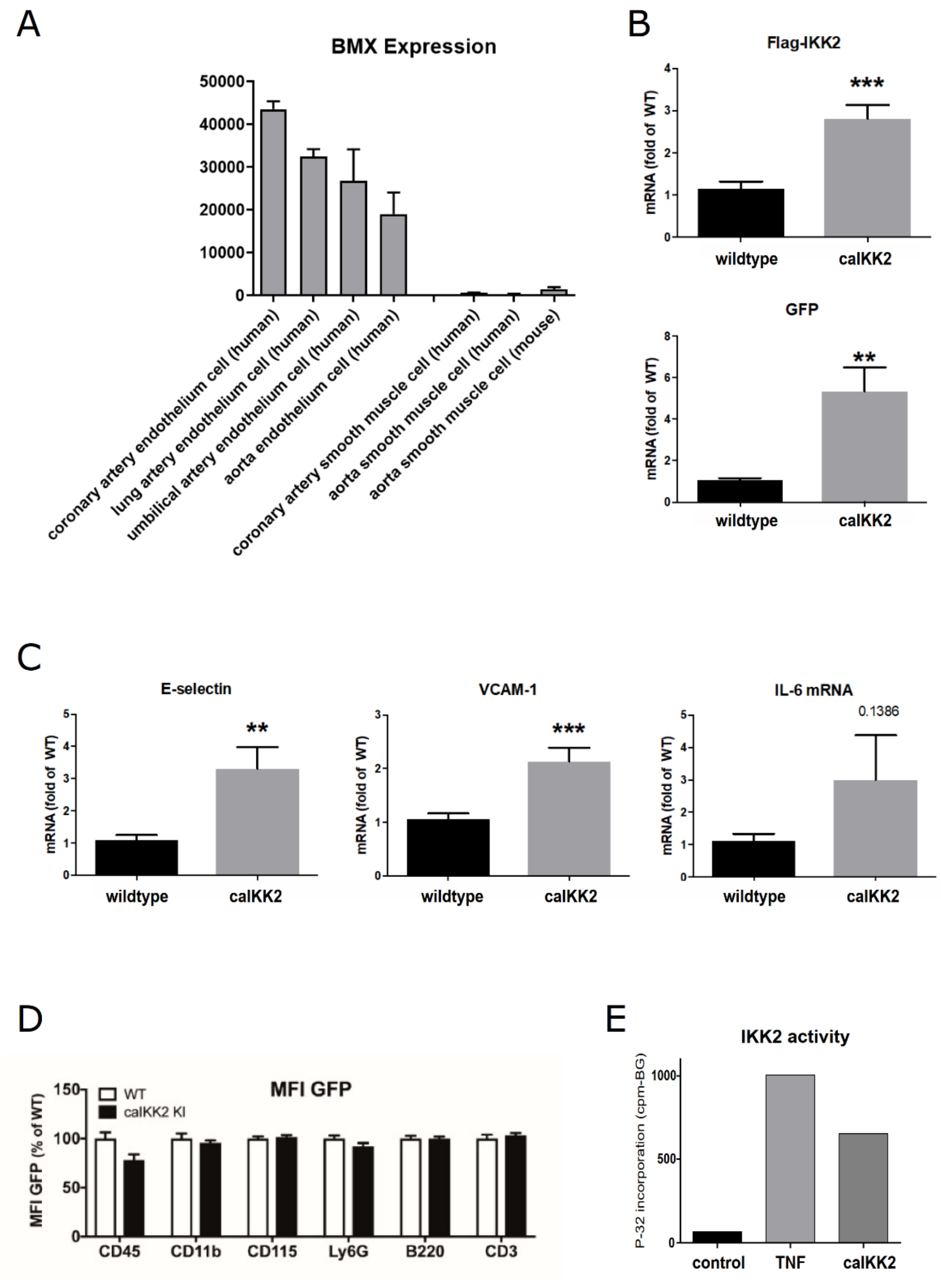

Supplementary Figure S1. Confirmatory data on the mouse model of arterial endothelial inflammation

A) Genevestigator database expression values for the BMX gene in various cell types as indicated (expression in endothelial but not in smooth muscle cells). B) Confirmation of the transgene expression (Flag-tagged constitutive active IKK2 and co-expressed GFP downstream of the IRES site) as assessed by reverse transcription-qPCR. C) Upregulation of inflammatory genes (E-selectin, VCAM-1 and IL-6)

in aortas expressing caIKK2. D) Lack of transgene expression in leukocytes of transgene mice (caIKK2 knock-in): Mean fluorescence intensity (MFI) of GFP is plotted compared to a wild-type (WT) control. E) IKK2 kinase activity of cells treated with TNFα and cells expressing the caIKK2 transgene as compared to control cells (as determined by a peptide kinase assay using the cognate IκB substrate peptide).

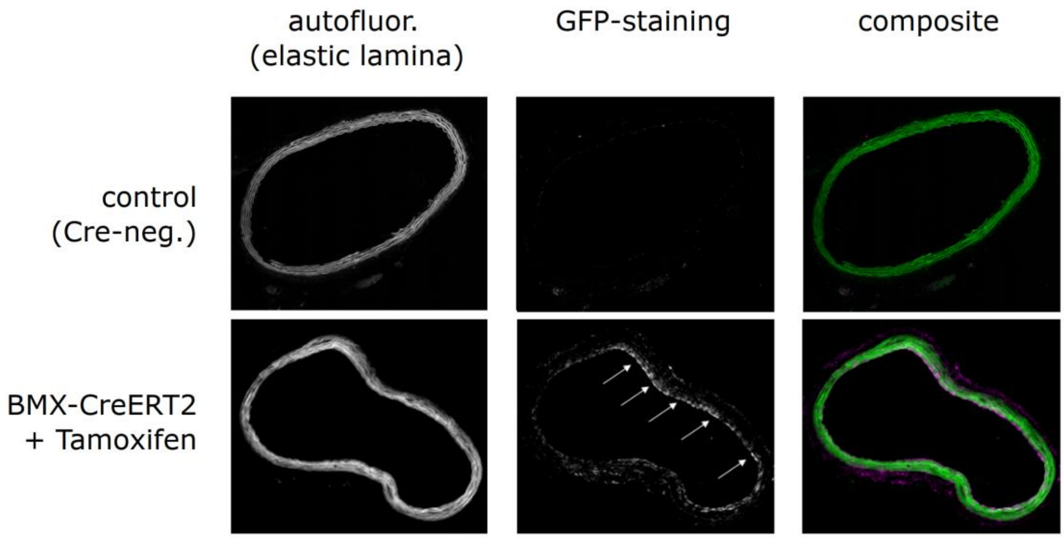

Supplementary Figure S2. Confirmation of the transgene expression in the endothelial layer after inducing the Cre recombinase activity by tamoxifen injection into mice.

Cre-negative control mice and BMX-CreERT2-positive mice (both containing the caIKK2-IRES-GFP transgene downstream of a loxP-flanked stop cassette) were injected five times (on 5 consecutive days) with tamoxifen as specified in the main Methods section, followed by isolation of aortae on day 10, fixation and paraffination. Paraffin sections were stained with anti-GFP antibodies (because intrinsic GFP fluorescence is lost by the paraffination procedure) and secondary antibodies coupled with a red-fluorescent dye (shown in magenta in the composite images). The internal elastic lamina (IEL) has a strong autofluorescence in the green channel – and serves as localization marker to specify the lumenal side of the aorta. Endothelial cells are located on the inner side, while smooth muscle cells are outside of the IEL.

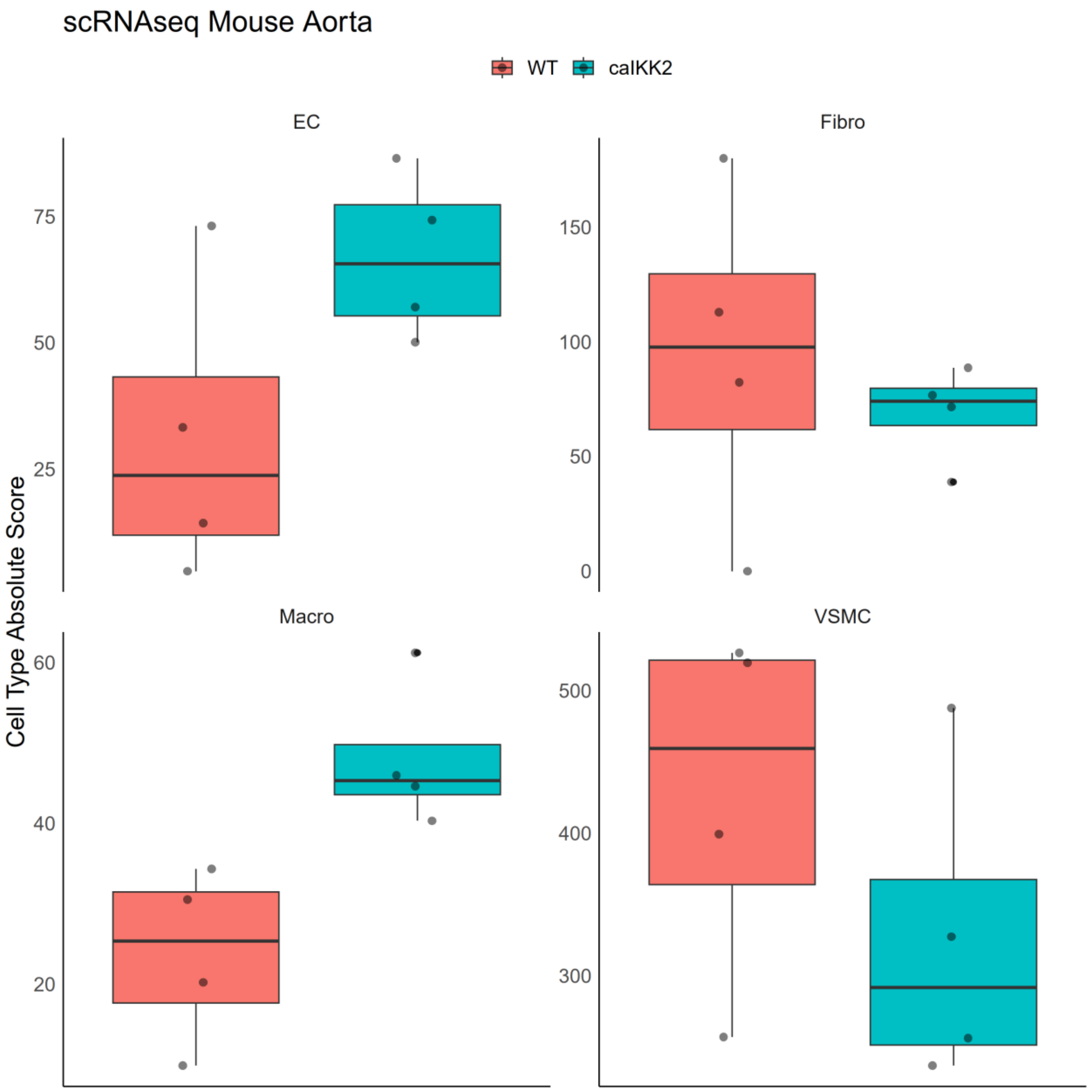

Supplementary Figure S3. Deconvolution of cell types from aorta samples using CIBERSORTx

Cell type signatures were determined for aortas from wild-type (WT) and transgene (caIKK2) mice with the "Impute Cell Fractions" module of CIBERSORTx, using the batch correction S-mode, in run mode absolute. All input files were in transcripts per million (tpm). Changes in cell profile expression upon endothelial caIKK2 expression are shown for endothelial cells (EC), macrophages (Macro), fibroblasts (Fibro) and vascular smooth muscle cells (VSMC).

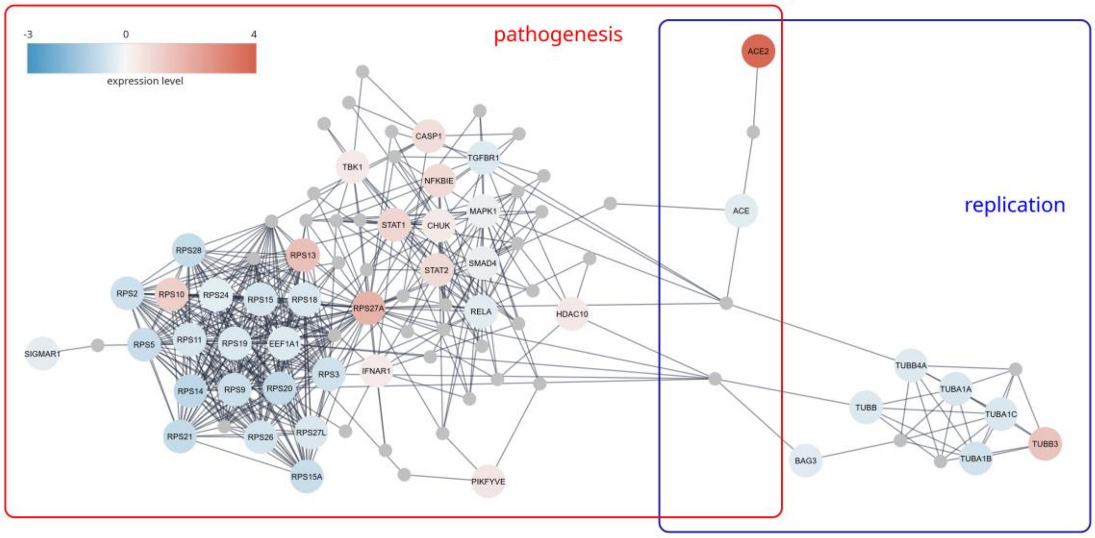

Supplementary Figure S4. Network analysis of differentially regulated genes after inflammatory activation of endothelial cells, which have roles in Coronavirus infection pathways.

Gene sets for *Coronavirus pathogenesis* (from Ingenuity Pathway Analysis) and for *Coronavirus replication* were uploaded separately to the *NetworkAnalyst* platform together with the expression levels of differentially regulated genes. The minimum *STRING* interactome networks that links these genes was computed for both gene sets. The resulting networks were downloaded as *graphml-files* and imported into the *Cytoscape* environment, where they were merged and analyzed for their overlap. ACE2, the highest upregulated gene upon inflammatory stimulation of arterial endothelial cells belongs to both pathways. Many of the virus pathogenesis genes are upregulated (in particular those, which have roles in inflammation) with the exception of several ribosomal protein genes. Genes of the replication pathway comprising mostly tubulin genes, important for transport and secretion of viral particles, were mostly downregulated (except for beta-tubulin).

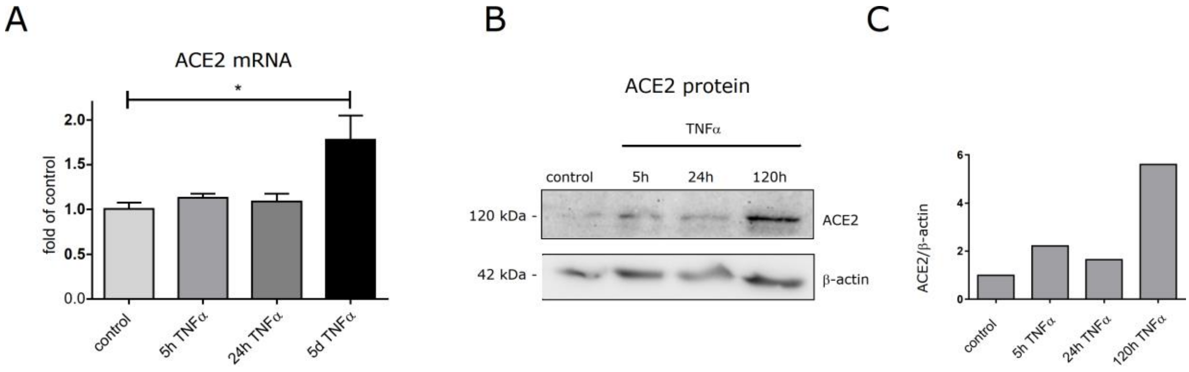

Supplementary Figure S5: ACE2 expression in HUVECs upon inflammatory activation

Human umbilical vein endothelial cells (HUVECs) were treated for different periods of time with TNFα (50 ng/ml), followed by RNA and protein extraction. A) RNA was reversed transcribed and ACE2 mRNA measured via quantitative PCR. B) Proteins were separated by SDS-PAGE and subjected to Western Blot analysis for ACE2 and β-actin protein expression. C) Quantification of the normalized ACE2 expression as shown in B) by densitometry. D), E) Quantification of late apoptotic/necrotic HUVECs treated with TNFα: HUVECs were treated with TNFα (50 ng/ml) for 5 days, trypsinized and stained with a cell death detection kit to assess Annexin V-binding, as well as uptake of 7-AAD by

permeable cells using flow cytometry. Late apoptotic/necrotic cells are positive for Annexin V as well as 7-AAD as indicated by the gates in the density plots (D). E) Quantification of these cells and statistical comparison (Students t-test, n=3).

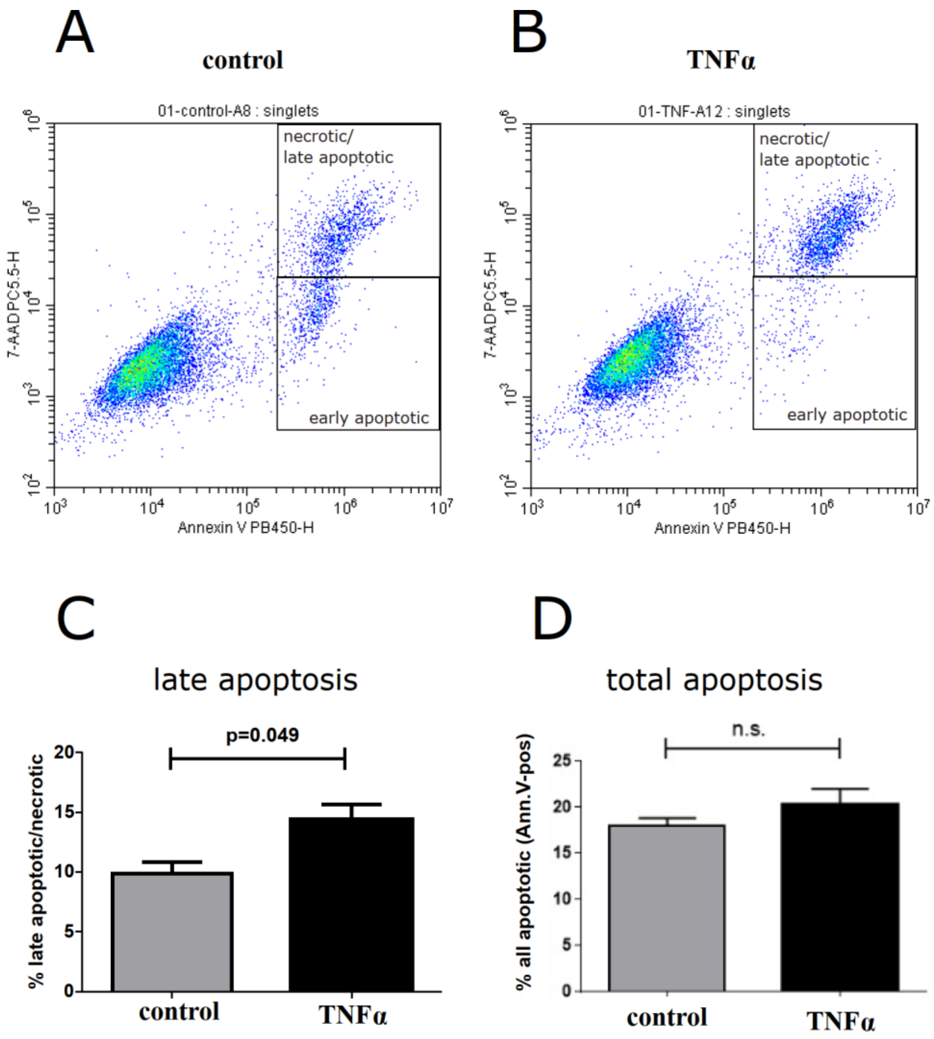

Suppl. Figure S6: Apoptosis in HUVECs

Isolated primary HUVECs were treated for 5 days with TNFα and the percentage of apoptotic cells (comprising early as well as late apoptosis/necrosis) was assessed in comparison to control cells using flow cytometry analysis of Annexin-V binding.

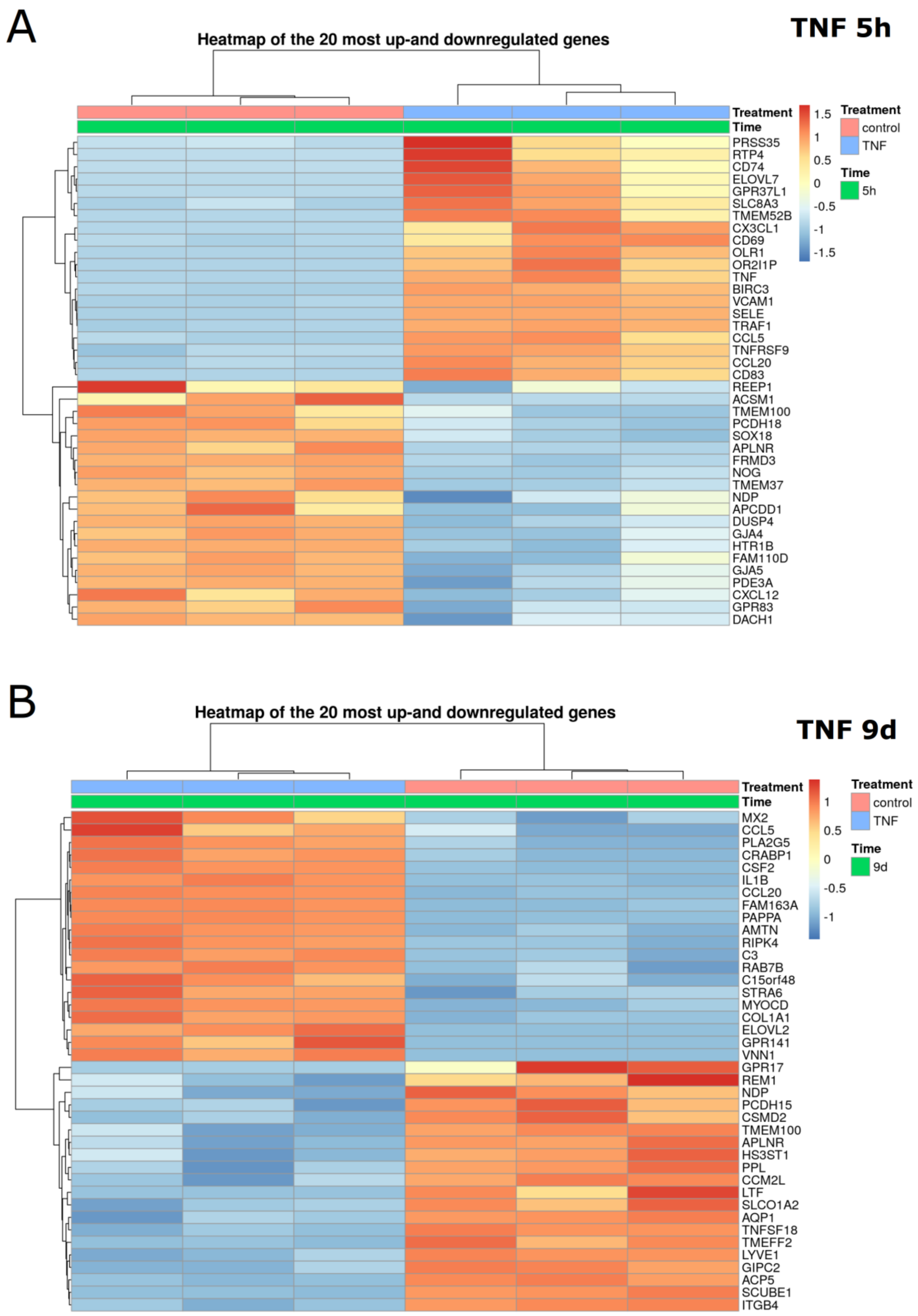

Suppl. Figure S7: Transcriptomic changes of HUVECs after short (5h) and long-term (9d) treatment with TNFα

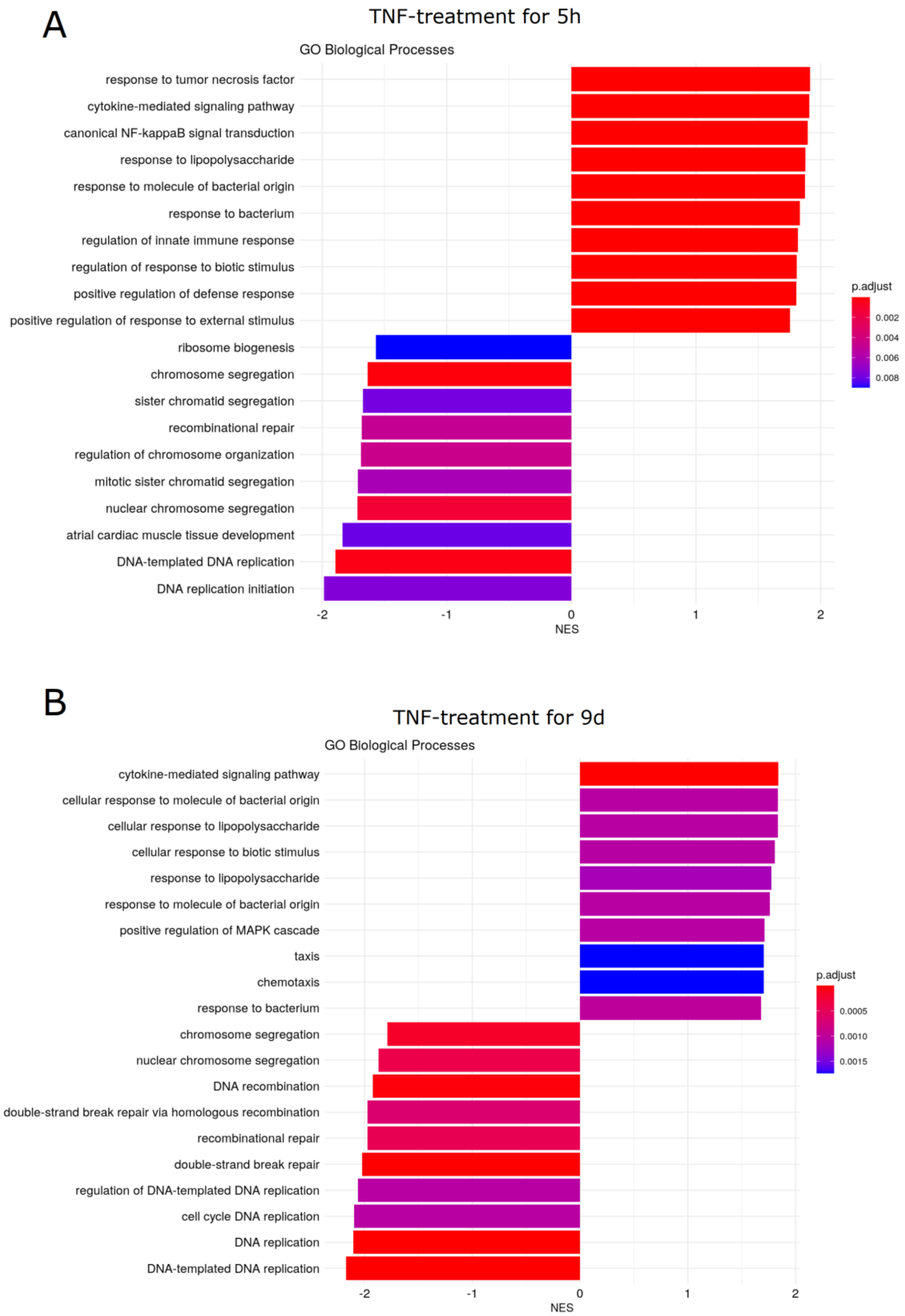

Suppl. Figure S8: Pathway analysis of differentially expressed genes in isolated human endothelial cells after prolonged treatment with TNFα:

HUVECs were treated for 5h or 9d with TNFα (50 ng/ml), followed by RNA extraction, sequencing and gene set enrichment analysis using the gene ontology of biological processes (n=3 for the different conditions).

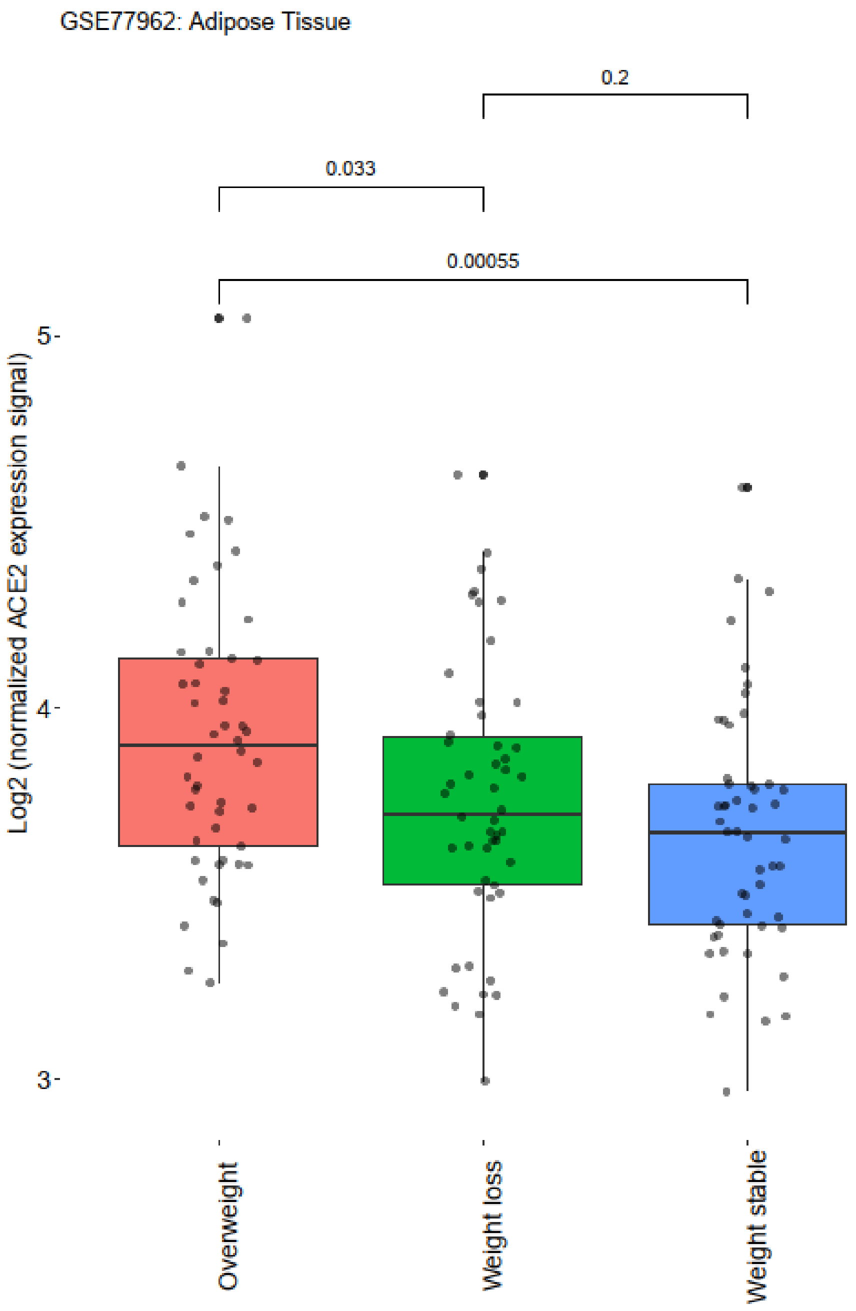

Suppl. Figure S9: Re-analysis of the dataset GSE77962 for ACE2 expression

ACE2 levels are highest in overweight humans, decline with weight loss and are lowest at a stable reduced weight (reference 30).

Suppl. Table S1: Comparison of the effects of endothelial caIKK2 expression in murine aortas of ApoE wild-type or knock-out background

caIKK2 on ApoE wildtype				caIKK2 on ApoE KO			
Symbols	logFC	adj.P.Val	Name	Symbols	logFC	adj.P.Val	Name
ACE2	3.761	9.15E-15	angiotensin I converting enzyme 2	Robo2	1.9736	6.0304E-10	roundabout guidance receptor 2
CORIN	3.196	1.04E-02	corin, serine peptidase	Ace2	1.8995	3.1442E-07	angiotensin I converting enzyme (peptidyl-dipeptidase A) 2
Serpina3g	2.594	8.15E-04	Ser (or Cyst) peptidase inhibitor, clade A, memb 3G	Serpina3f	1.705	0.000045798	"serine (or cysteine) peptidase inhibitor, clade A, member 3F"
GBP6	2.249	6.40E-06	guanylate binding protein family member 6	Gal	1.4617	0.00078753	galanin and GMAP prepropeptide
CXCL10	2.083	7.73E-03	C-X-C motif chemokine ligand 10	Serpina3g	1.4156	0.00025261	"serine (or cysteine) peptidase inhibitor, clade A, member 3G"
Clec2d	1.957	9.14E-08	C-type lectin domain family 2, member d	Serpina3i	1.3828	0.0010845	"serine (or cysteine) peptidase inhibitor, clade A, member 3I"
RPS27A	1.794	6.01E-05	ribosomal protein S27a	Grem1	1.3744	0.00050877	"gremlin 1, DAN family BMP antagonist"
Cxcl9	1.751	2.24E-03	chemokine (C-X-C motif) ligand 9	Cd55	1.3645	0.00023661	"CD55 molecule, decay accelerating factor for complement"
IGSF1	1.738	2.38E-02	immunoglobulin superfamily member 1	Abca13	1.3414	0.0022635	"ATP-binding cassette, sub-family A (ABC1), member 13"
CIITA	1.658	1.56E-02	class II major histocompatibility complex transact.	Gpr171	1.3132	0.00032853	G protein-coupled receptor 171
SBK2	1.657	3.07E-02	SH3 domain binding kinase family member 2	Cyp4f18	1.3003	0.0021674	"cytochrome P450, family 4, subfamily f, polypeptide 18"
GBP5	1.507	3.64E-03	guanylate binding protein 5	Lrrn3	1.257	0.0060936	"leucine rich repeat protein 3, neuronal"
CD55	1.499	6.34E-08	CD55 molecule (Cromer blood group)	Spdl1	1.2397	0.00024859	spindle apparatus coiled-coil protein 1
TNFSF10	1.468	5.15E-08	TNF superfamily member 10	Il18rap	1.2263	0.0014373	interleukin 18 receptor accessory protein
RPS13	1.451	3.21E-04	ribosomal protein S13	Prss30	1.2111	0.0033176	"protease, serine 30"
CD274	1.392	6.43E-03	CD274 molecule	Doc2b	1.2076	0.0010845	"double C2, beta"
HLA-A	1.386	5.84E-05	major histocompatibility complex, class I, A	Mmp13	1.2072	0.001086	matrix metallopeptidase 13
Tgtp1/Tgtp2	1.367	2.44E-02	T cell specific GTPase 1	Dmp1	1.2047	0.0099473	dentin matrix protein 1
TUBB3	1.347	2.79E-02	tubulin beta 3 class III	Mefv	1.1897	0.0001645	Mediterranean fever
IL2RG	1.321	2.30E-04	interleukin 2 receptor subunit gamma	Mir155hg	1.1888	0.01058	Mir155 host gene (non-protein coding)
CCNL1	1.281	4.61E-10	cyclin L1	Serpina3n	1.1444	0.000026197	"serine (or cysteine) peptidase inhibitor, clade A, member 3N"
NOD2	1.237	8.37E-06	nucleotide binding oligomer. domain containing 2	Spint1	1.1418	0.00032853	"serine protease inhibitor, Kunitz type 1"
NLRC5	1.236	1.06E-02	NLR family CARD domain containing 5	B3gnt3	1.1338	0.001126	"UDP-GlcNAc:betaGal beta-1,3-N-acetylglucosaminyltransferase 3"
TCIM	1.232	3.74E-03	transcriptional and immune response regulator	Ercc6l	1.1326	0.0015462	
DNAJC28	1.195	1.06E-02	DnaJ heat shock protein family (Hsp40) member C28	Atp8b4	1.1313	0.00010188	"ATPase, class I, type 8B, member 4"

caIKK2 on ApoE wildtype				caIKK2 on ApoE KO			
**Symbols**	**logFC**	**adj.P.Val**	**Name**	**Symbols**	**logFC**	**adj.P.Val**	**Name**
SCX	-0.77	4.40E-02	scleraxis bHLH transcription factor	Trnp1	-1.0423	0.0057612	TMF1-regulated nuclear protein 1
RPS5	-0.773	2.66E-07	ribosomal protein S5	5430431A17Rik	-1.0477	0.025495	RIKEN cDNA 5430431A17 gene
ATF5	-0.774	3.97E-10	activating transcription factor 5	Smim5	-1.0517	0.0016514	small integral membrane protein 5
RPL37A	-0.782	1.82E-06	ribosomal protein L37a	Lrrn1	-1.0724	0.023821	"leucine rich repeat protein 1, neuronal"
TRNP1	-0.799	1.25E-02	TMF1 regulated nuclear protein 1	Sost	-1.0732	0.004652	sclerostin
TMEM45A	-0.805	1.60E-03	transmembrane protein 45A	Ppp1r14c	-1.0834	0.00546	"protein phosphatase 1, regulatory inhibitor subunit 14C"
RPS28	-0.812	1.51E-05	ribosomal protein S28	Mir143	-1.1041	0.020315	microRNA 143
TMEM98	-0.813	4.01E-04	transmembrane protein 98	Cck	-1.122	0.0080187	cholecystokinin
PERP	-0.82	2.07E-03	p53 apoptosis effector related to PMP22	Slc22a2	-1.1309	0.014117	"solute carrier family 22 (organic cation transporter), member 2"
RBP1	-0.821	3.08E-04	retinol binding protein 1	Adra2a	-1.1322	0.0014748	"adrenergic receptor, alpha 2a"
TMEM160	-0.823	3.34E-04	transmembrane protein 160	Mylk4	-1.1365	0.0025078	"myosin light chain kinase family, member 4"
Ppbp	-0.824	2.67E-02	pro-platelet basic protein	Klk10	-1.1593	0.010717	kallikrein related-peptidase 10
C1orf198	-0.839	2.07E-02	chromosome 1 open reading frame 198	Gja6	-1.1628	0.0045197	"gap junction protein, alpha 6"
RPS20	-0.84	2.16E-07	ribosomal protein S20	Col19a1	-1.1733	0.0068681	"collagen, type XIX, alpha 1"
RPS21	-0.849	9.87E-08	ribosomal protein S21	Cdh16	-1.1803	0.010639	cadherin 16
RPL39	-0.859	1.47E-06	ribosomal protein L39	4930417O22Rik	-1.1817	0.0077992	RIKEN cDNA 4930417O22 gene
EFHD1	-0.881	2.87E-03	EF-hand domain family member D1	Ky	-1.1937	0.0012464	kyphoscoliosis peptidase
KRT18	-0.889	7.14E-05	keratin 18	Ncan	-1.2095	0.0090516	neurocan
TENT5B	-0.899	1.60E-02	terminal nucleotidyltransferase 5B	Fam25c	-1.2294	0.0083178	"family with sequence similarity 25, member C"
KCNJ15	-0.937	1.60E-02	potassium inwardly rectifying channel subfamily J15	Kcnc4	-1.2363	0.0055485	"potassium voltage gated channel, Shaw-related subfamily, member 4"
RPS14	-0.944	1.04E-06	ribosomal protein S14	Rab17	-1.2706	0.0010468	"RAB17, member RAS oncogene family"
CREB3L1	-0.971	9.01E-05	cAMP responsive element binding protein 3 like 1	Casq1	-1.3696	0.0005563	calsequestrin 1
Rplp1	-1.012	8.38E-10	ribosomal protein, large, P1	Ihh	-1.4414	0.0005229	Indian hedgehog
CLDN3	-1.1	4.57E-03	claudin 3	Slc22a1	-1.4598	0.0004883	"solute carrier family 22 (organic cation transporter), member 1"
NPAS2	-1.274	2.18E-02	neuronal PAS domain protein 2	Lsmem1	-1.4844	0.0009313	leucine-rich single-pass membrane protein 1

Suppl. Table S2: All biological functions altered in murine aortas after inflammatory activation of endothelial cells on ApoE-wild-type background as assessed by Ingenuity Pathway Analysis

Categories	Diseases or Functions Annotation	p-value	Predicted Activation State	Activation z-score	Molecules	# Molecules
Protein Synthesis	Initiation of translation of protein	4,48E-22		0,294	AGO2,EIF2AK4,EIF2S2,EIF3E,EIF3F,EIF3H,EIF3I,EIF3K,EIF4A2,EIF4EBP2,EIF4H,FMR1,IMPACT,MTIF2,MY	56
RNA Damage and Repair	Nonsense-mediated mRNA decay	1,59E-18			EIF3E,PPP2CA,PPP2R1A,RPL10,RPL12,RPL13,RPL14,RPL18,RPL18A,RPL22,RPL23,RPL26,RPL35,RPL35A,	44
Cancer,Cell Death and Survival,Organismal Injury and Abnormalities,Tumor Morphology	Cell death of osteosarcoma cells	9,06E-15	Increased	3,429	CAPN6,CELA1,EEF1A1,EIF3E,EIF3F,HSPA9,NUP155,NUP214,PHF5A,PSMA5,PSMB1,PSMB5,RAN,RPL10,	46
Protein Synthesis	Translation	3,30E-14		-0,078	AGO1,AGO2,AKAP8L,AKT1,ATF5,CHCHD1,CNBP,DHX9,EEF1A1,EEF1B2,EEF1D,EEF2,EIF2AK4,EIF2S2,EIF	96
Protein Synthesis	Translation of protein	4,22E-12		0,656	AGO1,AGO2,AKAP8L,AKT1,ATF5,CHCHD1,CNBP,DHX9,EEF1A1,EIF2AK4,EIF2S2,EIF3E,EIF3F,EIF3H,EIF3I,	89
Protein Synthesis	Metabolism of protein	5,21E-12		-1,613	ACE,ACE2,ADAM12,ADRM1,AFG3L2,AGO1,AGO2,AHSG,AKAP8L,AKT1,AMFR,ANAPC10,ANAPC11,ATF5	237
Protein Synthesis	Synthesis of protein	1,10E-11		-1,35	ADRM1,AGO1,AGO2,AKAP8L,AKT1,ATF5,BCR,CAV1,CDKN1B,CHCHD1,CIRBP,CNBP,CYP1B1,DHX9,EEF1	125
Protein Synthesis	Expression of protein	4,93E-10		0,33	ADRM1,AGO1,AGO2,AKAP8L,AKT1,ATF5,BCR,CAV1,CDKN1B,CHCHD1,CNBP,DHX9,EEF1A1,EIF2AK4,EIF	97
Cancer,Cell Death and Survival,Organismal Injury and Abnormalities	Cell death of malignant tumor	4,14E-08	Increased	3,255	AFAP1L2,AIFM1,AKT1,AKT1S1,BAD,BCL2L1,BRD2,CAPN6,CASP1,CAT,Cdc42,CDKN1B,CELA1,EEF1A1,EIF	84
Cancer,Cell Death and Survival,Organismal Injury and Abnormalities,Tumor Morphology	Necrosis of malignant tumor	7,05E-08	Increased	3,255	AFAP1L2,AIFM1,AKT1,AKT1S1,BAD,BCL2L1,BRD2,CAPN6,CASP1,CAT,Cdc42,CDKN1B,CELA1,EEF1A1,EIF	83
Cancer,Cell Death and Survival,Organismal Injury and Abnormalities,Tumor Morphology	Cell death of cancer cells	1,19E-07	Increased	3,255	AFAP1L2,AIFM1,AKT1,AKT1S1,BAD,BCL2L1,BRD2,CAPN6,CASP1,CAT,Cdc42,CDKN1B,CELA1,EEF1A1,EIF	82
Cancer,Cell Death and Survival,Organismal Injury and Abnormalities,Tumor Morphology	Cell death of tumor cells	2,50E-07	Increased	3,27	ADAM12,AFAP1L2,AIFM1,AKT1,AKT1S1,BAD,BCL2L1,BRD2,CAPN6,CASP1,CAT,CAV1,CCND3,CD274,Cd	94
Cancer,Cell Death and Survival,Organismal Injury and Abnormalities	Cell death of tumor	2,89E-07	Increased	3,27	ADAM12,AFAP1L2,AIFM1,AKT1,AKT1S1,BAD,BCL2L1,BRD2,CAPN6,CASP1,CAT,CAV1,CCND3,CD274,Cd	96
Cancer,Cell Death and Survival,Organismal Injury and Abnormalities,Tumor Morphology	Necrosis of tumor	3,91E-07	Increased	3,27	ADAM12,AFAP1L2,AIFM1,AKT1,AKT1S1,BAD,BCL2L1,BRD2,CAPN6,CASP1,CAT,CAV1,CCND3,CD274,Cd	95
Gene Expression,Protein Synthesis	Translation of mRNA	5,39E-07		0	AGO2,AKT1,EIF2AK4,EIF3E,EIF3F,EIF3H,EIF3I,EIF3K,EIF4A2,EIF4EBP2,EIF4H,FMR1,FOXO3,ILF3,IMPACT,	39
Gene Expression	Expression of mRNA	2,93E-06		-0,696	AGO2,AKT1,COPZ1,DDX5,DHX9,EIF2AK4,EIF3E,EIF3F,EIF3H,EIF3I,EIF3K,EIF4A2,EIF4EBP2,EIF4H,FMR1,F	53
Cell Death and Survival	Necrosis	1,04E-05		1,669	ABCC9,ACAT1,ACE,ACE2,ACVRL1,ADAM12,ADRA1A,ADRM1,AFAP1L2,AGO1,AIFM1,AKT1,AKT1S1,ALKB	435
Cell Death and Survival	Cell death of sarcoma cell lines	2,61E-05		0,803	AIFM1,AKT1,ATAD2,BAD,BCL2L1,BCLAF1,BMX,BUB3,CABLES2,CAPNS1,CAV1,CDKN1B,COP1,DHX9,DNA	50
Hereditary Disorder,Organismal Injury and Abnormalities,Skeletal and Muscular Disorders	Hereditary myopathy	3,33E-05			ABCC9,ACE,ADRA1A,AIFM1,AP4S1,AP5B1,ASPN,BAG3,BCL2L1,BGN,CASQ1,CCND3,CELF2,CEP85L,CFL2,	110
Cell Death and Survival	Cell death of cervical cancer cell lines	8,52E-05	Increased	2,054	ADRM1,AIFM1,AKT1,ANXA5,ATN1,BAD,BCL2L1,BUB3,CASP1,CAT,CCAR2,CCND3,CCT4,CDKN1B,CENPB,	75
Developmental Disorder,Hereditary Disorder,Neurological Disease,Organismal Injury and Abnormaliti	Cerebro-oculo-facio-skeletal syndrome	1,04E-04			ERCC1,ERCC2,ERCC5,ERCC6	4
Cell Death and Survival	Apoptosis of cervical cancer cell lines	1,22E-04	Increased	2,424	AIFM1,AKT1,ANXA5,BAD,BCL2L1,CASP1,CAT,CCAR2,CCND3,CCT4,CDKN1B,CENPB,DNAJA3,DNM1L,DY	60
Cellular Assembly and Organization	Regulation of mitochondria	1,27E-04			BRCA2,EEF2,ITGB3,MYC,NUP214,PPP2CA,PPP2R1A,RAN,TUBA1B,ZBTB33	10
Cell Death and Survival	Cell death of bone cancer cell lines	1,47E-04		0,546	AIFM1,AKT1,ATAD2,BCL2L1,BCLAF1,BUB3,CABLES2,CAPNS1,CAV1,CDKN1B,COP1,DHX9,DNAJA3,EEF1	39
Gene Expression	Expression of RNA	1,58E-04		0,818	ACE,ACTG1,ACTR3,ACVRL1,AFAP1L2,AGO1,AGO2,AKAP8L,AKT1,ANGPT1,ARF4,ASXL2,ATAD2,ATF5,AT	346
Cancer,Endocrine System Disorders,Organismal Injury and Abnormalities,Reproductive System Diseas	Stage III-IV serous ovarian adenocarcinoma	1,59E-04			CD274,KLK10,PARP2,PARP3,TUBA1A,TUBA1C,TUBB3,TUBB4A,VEGFA	9
Cellular Assembly and Organization,DNA Replication, Recombination, and Repair	Formation of nuclear envelope	1,93E-04		-0,342	PPP2CA,PPP2R1A,RAN,REEP5,RTN1,RTN2,RTN3	7
Cell Death and Survival	Apoptosis of bone cancer cell lines	2,02E-04		1,221	AIFM1,AKT1,ATAD2,BCL2L1,BCLAF1,BUB3,CABLES2,CAPNS1,CAV1,CDKN1B,DNAJA3,EEF1A1,ETHE1,FB	33
Cell Death and Survival	Apoptosis of sarcoma cell lines	2,13E-04		1,069	AIFM1,AKT1,ATAD2,BAD,BCL2L1,BCLAF1,BMX,BUB3,CABLES2,CAPNS1,CAV1,CDKN1B,DNAJA3,EEF1A1,	40
Gene Expression,Protein Synthesis	Initiation of translation of mRNA	2,48E-04		-0,063	AGO2,EIF2AK4,EIF3E,EIF3F,EIF3H,EIF3I,EIF3K,EIF4A2,EIF4EBP2,EIF4H,FMR1,IMPACT,MTIF2,TPR	14
Cancer,Cardiovascular Disease,Developmental Disorder,Hematological Disease,Hereditary Disorder,O	Diamond-Blackfan anemia	2,58E-04			RPL18,RPL26,RPL35,RPL35A,RPS10,RPS15A,RPS19,RPS20,RPS24,RPS26,RPS28	11
Developmental Disorder,Hereditary Disorder,Organismal Injury and Abnormalities,Skeletal and Musc	Emery-Dreifuss muscular dystrophy	2,74E-04			ASPN,BGN,CELF2,COL6A1,COL6A2,CYB561,EMD,LOXL1,LTBP3,PLOD2,RASSF1,SDC2,SDCBP,SUN2,SYNE	16
Cancer,Organismal Injury and Abnormalities	Peritoneal carcinoma	2,85E-04			ATIC,CD274,CHD1,IFNGR1,MAP2K2,MAPK1,PARP2,PARP3,TUBA1A,TUBA1C,TUBB3,TUBB4A,VEGFA	13
Cancer,Endocrine System Disorders,Organismal Injury and Abnormalities,Reproductive System Diseas	Advanced ovarian carcinosarcoma	2,93E-04			CD274,PARP2,PARP3,TUBA1A,TUBA1C,TUBB3,TUBB4A,VEGFA	8
Cellular Movement	Invasion of breast cell lines	2,95E-04	Decreased	-2,11	AKT1,ARF4,CDKN1B,CSNK2B,EIF3E,FBLN5,FOXO3,GIT2,HRAS,ITGB3,MAP2K2,MAPK1,OGT,PARVA,RAC1	20
Infectious Diseases	Replication of SARS coronavirus	3,13E-04		-0,816	ACE2,BAG3,IFNAR1,PIKFYVE,SIGMAR1,STAT1	6
Cancer,Organismal Injury and Abnormalities,Reproductive System Disease	Recurrent fallopian tube serous adenocarcinoma	3,46E-04			MAP2K2,MAPK1,PARP2,PARP3,TUBA1A,TUBA1C,TUBB3,TUBB4A,VEGFA	9
Cancer,Endocrine System Disorders,Organismal Injury and Abnormalities,Reproductive System Diseas	Ovarian carcinosarcoma	3,60E-04			BRCA2,CD274,PARP2,PARP3,TET2,TUBA1A,TUBA1C,TUBB3,TUBB4A,VEGFA	10
Cell Death and Survival	Cell death of connective tissue cells	3,97E-04		0,532	ADAM12,AIFM1,AKT1,ATF5,ATN1,BAD,BCL2L1,BGN,BRCA2,C1orf159,CA3,CABLES2,CAPNS1,CASP1,CA	109
Cancer,Organismal Injury and Abnormalities,Reproductive System Disease	Invasive breast adenocarcinoma	4,10E-04			AKT1,BCCIP,BCL2L1,BGN,BRCA2,CCND3,CD274,CHD1,ELP3,HSPB8,LGALS1,LTBP4,MAP2K2,MKI67,MYC,	30
Cancer,Endocrine System Disorders,Organismal Injury and Abnormalities,Reproductive System Diseas	Advanced high-grade serous ovarian cancer	4,45E-04			CD274,PARP2,PARP3,TUBA1A,TUBA1C,TUBB3,TUBB4A,VEGFA	8
Cancer,Organismal Injury and Abnormalities,Reproductive System Disease	Fallopian tube carcinosarcoma	4,45E-04			CD274,PARP2,PARP3,TUBA1A,TUBA1C,TUBB3,TUBB4A,VEGFA	8
Cancer,Organismal Injury and Abnormalities,Reproductive System Disease	Primary peritoneal carcinosarcoma	4,45E-04			CD274,PARP2,PARP3,TUBA1A,TUBA1C,TUBB3,TUBB4A,VEGFA	8
Gene Expression	Initiation of expression of RNA	4,52E-04		-0,721	AGO2,EIF2AK4,EIF3E,EIF3F,EIF3H,EIF3I,EIF3K,EIF4A2,EIF4EBP2,EIF4H,ERCC2,FMR1,IMPACT,MAZ,MED4	40
Cell Cycle,Renal and Urological System Development and Function	Arrest in G2/M phase of kidney cell lines	4,54E-04			OGT,PPP2CA,PPP2CB,RASSF1,RBBP6	5
Cell Death and Survival	Cell death of schwannoma cell lines	4,54E-04		1,687	CDKN1B,MYC,RABL6,TNFRSF10A,TNFSF10	5
Cell Cycle,Hair and Skin Development and Function	Arrest in G2/M phase of epithelial cell lines	4,54E-04			OGT,PPP2CA,PPP2CB,RASSF1,RBBP6	5
Gene Expression	Transcription	4,56E-04		1,034	ACE,ACTG1,ACTR3,ACVRL1,AFAP1L2,AGO1,AGO2,AKAP8L,AKT1,ANGPT1,ARF4,ASXL2,ATAD2,ATF5,AT	323
Nucleic Acid Metabolism,Small Molecule Biochemistry	Methylation of cytosine	4,80E-04			IDH1,IDH2,TET2,TRIM28	4
Cancer,Organismal Injury and Abnormalities,Reproductive System Disease	Invasive HER2 negative hormone receptor negati	4,91E-04			CD274,CHD1,PARP2,PARP3,TUBA1A,TUBA1C,TUBB,TUBB3,TUBB4A,VEGFA	10
Protein Degradation,Protein Synthesis	Catabolism of protein	4,97E-04		-0,688	ACE,ACE2,ADAM12,ADRM1,AFG3L2,AKT1,AMFR,ANAPC10,ANAPC11,ATP1B2,ATP1B3,AZIN1,BACE2,B	113
Small Molecule Biochemistry	Metabolism of 2-oxoglutaric acid	5,73E-04			ADHFE1,DLD,IDH1,IDH3B,IDH3G,PHYH	6
Amino Acid Metabolism,Small Molecule Biochemistry	Catabolism of branched chain amino acids	6,56E-04			ACAD8,ACAT1,DBT,DLD,IVD,MCCC1,MCCC2,PPM1K	8
Cancer,Endocrine System Disorders,Organismal Injury and Abnormalities,Reproductive System Diseas	Stage IIIC-IV serous ovarian cancer	6,56E-04			KLK10,PARP2,PARP3,TUBA1A,TUBA1C,TUBB3,TUBB4A,VEGFA	8
Cancer,Organismal Injury and Abnormalities	Stage III-IV primary peritoneal cancer	6,56E-04			CD274,PARP2,PARP3,TUBA1A,TUBA1C,TUBB3,TUBB4A,VEGFA	8
Cancer,Endocrine System Disorders,Organismal Injury and Abnormalities,Reproductive System Diseas	Stage III serous ovarian adenocarcinoma	6,56E-04			KLK10,PARP2,PARP3,TUBA1A,TUBA1C,TUBB3,TUBB4A,VEGFA	8
Cell Death and Survival	Apoptosis	8,73E-04		1,602	ACE,ACE2,ACIN1,ACVRL1,ADAM12,ADRA1A,AFAP1L2,AGO1,AGO2,AIFM1,AIFM2,AKAP8,AKT1,AKT1S1,	406
Cancer,Organismal Injury and Abnormalities	Primary recurrent peritoneal carcinoma	8,74E-04			CD274,MAP2K2,MAPK1,PARP2,PARP3,TUBA1A,TUBA1C,TUBB3,TUBB4A,VEGFA	10
Cancer,Endocrine System Disorders,Organismal Injury and Abnormalities,Reproductive System Diseas	Recurrent serous ovarian carcinoma	8,74E-04			CD274,MAP2K2,MAPK1,PARP2,PARP3,TUBA1A,TUBA1C,TUBB3,TUBB4A,VEGFA	10
Cell Cycle,Gene Expression	Binding of PPAR response element	9,36E-04		-0,218	FOXO1,HRAS,RXRA,THRA,TXN	5
Dermatological Diseases and Conditions,Organismal Injury and Abnormalities	UV-sensitive syndrome	9,36E-04			ERCC1,ERCC2,ERCC5,ERCC6,UVSSA	5
Respiratory System Development and Function	Elastance of lung	9,36E-04		-0,555	ACE2,CAV1,CELA1,MFAP4,SELENON	5
Cancer,Organismal Injury and Abnormalities,Reproductive System Disease	Stage III-IV fallopian tube cancer	9,40E-04			CD274,PARP2,PARP3,TUBA1A,TUBA1C,TUBB3,TUBB4A,VEGFA	8
Cardiovascular System Development and Function,Cell Morphology,Embryonic Development,Organ D	Size of cardiomyocytes	9,46E-04			AKT1,CAPNS1,FOXO1,FOXO3,FTO,HRAS,LIMS1,MAP3K1,MYC,NPR1,RAPGEF3,RASSF1,RBP4,RGS2,RHEE	17
Cancer,Organismal Injury and Abnormalities,Reproductive System Disease	Hormone receptor positive breast adenocarcinon	1,00E-03			AKT1,CD274,CHD1,FKBP1A,PARP2,PARP3,TUBA1A,TUBA1C,TUBB3,TUBB4A,VEGFA	11
Cell Cycle	Polyploidy	1,00E-03		0,869	Anp32b,CCP110,CKS1B,ERCC1,FAN1,FBXO5,H3-3A/H3-3B,IRF1,SAE1,STAT1,TRIM24	11

Categories	Diseases or Functions Annotation	p-value	Predicted Activation State	Activation z-score	Molecules	# Molecules
Nucleic Acid Metabolism,Small Molecule Biochemistry	5-hydroxymethylation of cytosine	1,03E-03			IDH1,IDH2,TET2	3
Cell-To-Cell Signaling and Interaction	Adhesion of Streptococcus pneumoniae D39	1,03E-03			CD151,CD63,CD9	3
Cell Morphology	Polarity of eye cell lines	1,03E-03			MAPRE1,MAPRE3,RASSF1	3
Cell-To-Cell Signaling and Interaction	Adhesion of Neisseria meningitidis strain MC58 C	1,03E-03			CD151,CD63,CD9	3
Cell Signaling,Nucleic Acid Metabolism,Small Molecule Biochemistry	Incorporation of guanosine 5'-O-(3-thiotriphosph	1,03E-03			CXCL10,GNAI2,GNAI3	3
Connective Tissue Development and Function	Development of fat pad	1,03E-03			CAV1,SEMA7A,VEGFA	3
Cell-To-Cell Signaling and Interaction	Adhesion of Neisseria lactamica NL1009	1,03E-03			CD151,CD63,CD9	3
Cardiovascular System Development and Function,Cellular Development,Cellular Growth and Prolifer	Colony formation of endothelial cell lines	1,03E-03			MYC,RHOC,SF3B1	3
Cancer,Organismal Injury and Abnormalities	Primary peritoneal serous adenocarcinoma	1,14E-03			CD274,MAP2K2,MAPK1,PARP2,PARP3,TUBA1A,TUBA1C,TUBB3,TUBB4A,VEGFA	10
Cardiovascular Disease,Developmental Disorder,Hematological Disease,Organismal Injury and Abnorr	Congenital aplastic anemia	1,19E-03			BRCA2,CAT,FKBP1A,GPX1,RPL18,RPL26,RPL35,RPL35A,RPS10,RPS15A,RPS19,RPS20,RPS24,RPS26,RPS2	16
Cancer,Organismal Injury and Abnormalities	Stage III primary peritoneal cancer	1,25E-03			PARP2,PARP3,TUBA1A,TUBA1C,TUBB3,TUBB4A,VEGFA	7
Cancer,Organismal Injury and Abnormalities	Advanced primary BRCA2 mutation positive perit	1,25E-03			CD274,PARP2,PARP3,TUBA1A,TUBA1C,TUBB3,TUBB4A	7
Cancer,Organismal Injury and Abnormalities	Advanced primary BRCA1 mutation positive perit	1,25E-03			CD274,PARP2,PARP3,TUBA1A,TUBA1C,TUBB3,TUBB4A	7
Cancer,Endocrine System Disorders,Organismal Injury and Abnormalities,Reproductive System Diseas	Stage IV serous ovarian adenocarcinoma	1,25E-03			PARP2,PARP3,TUBA1A,TUBA1C,TUBB3,TUBB4A,VEGFA	7
Cancer,Organismal Injury and Abnormalities	Stage IV primary peritoneal cancer	1,25E-03			PARP2,PARP3,TUBA1A,TUBA1C,TUBB3,TUBB4A,VEGFA	7
Cancer,Endocrine System Disorders,Organismal Injury and Abnormalities,Reproductive System Diseas	Advanced BRCA2 mutation positive ovarian tumo	1,25E-03			CD274,PARP2,PARP3,TUBA1A,TUBA1C,TUBB3,TUBB4A	7
Cancer,Endocrine System Disorders,Organismal Injury and Abnormalities,Reproductive System Diseas	BRCA2 mutation positive ovarian carcinosarcoma	1,25E-03			CD274,PARP2,PARP3,TUBA1A,TUBA1C,TUBB3,TUBB4A	7
Cancer,Organismal Injury and Abnormalities,Reproductive System Disease	BRCA2 mutation positive fallopian tube carcinosa	1,25E-03			CD274,PARP2,PARP3,TUBA1A,TUBA1C,TUBB3,TUBB4A	7
Cancer,Organismal Injury and Abnormalities,Reproductive System Disease	Stage III fallopian tube cancer	1,25E-03			PARP2,PARP3,TUBA1A,TUBA1C,TUBB3,TUBB4A,VEGFA	7
Cancer,Endocrine System Disorders,Organismal Injury and Abnormalities,Reproductive System Diseas	BRCA1 mutation positive ovarian carcinosarcoma	1,25E-03			CD274,PARP2,PARP3,TUBA1A,TUBA1C,TUBB3,TUBB4A	7
Cancer,Organismal Injury and Abnormalities,Reproductive System Disease	Primary BRCA2 mutation positive peritoneal carci	1,25E-03			CD274,PARP2,PARP3,TUBA1A,TUBA1C,TUBB3,TUBB4A	7
Cancer,Organismal Injury and Abnormalities,Reproductive System Disease	BRCA1 mutation positive fallopian tube carcinosa	1,25E-03			CD274,PARP2,PARP3,TUBA1A,TUBA1C,TUBB3,TUBB4A	7
Cancer,Endocrine System Disorders,Organismal Injury and Abnormalities,Reproductive System Diseas	Advanced BRCA1 mutation positive ovarian tumo	1,25E-03			CD274,PARP2,PARP3,TUBA1A,TUBA1C,TUBB3,TUBB4A	7
Cancer,Organismal Injury and Abnormalities,Reproductive System Disease	Primary BRCA1 mutation positive peritoneal carci	1,25E-03			CD274,PARP2,PARP3,TUBA1A,TUBA1C,TUBB3,TUBB4A	7
Cellular Development,Reproductive System Development and Function	Epithelial-mesenchymal transition of breast cell li	1,26E-03		-0,025	AKT1,CDKN1B,CSNK2B,CUL4A,EIF3E,FBLN5,HRAS,IDH1,ITGB3,MAP2K2,MAPK1,PCBP1,RAC1,SEMA7A,T	15
Cell Death and Survival	Apoptosis of T-cell hybrid cells	1,26E-03		-0,863	AKT1,BCL2L1,DNAJA3,HRAS,MYC,RELA,RORC,SIVA1,TNFAIP3	9
Cancer,Endocrine System Disorders,Organismal Injury and Abnormalities,Reproductive System Diseas	Platinum-sensitive recurrent ovarian cancer	1,26E-03			BRCA2,CD274,PARP2,PARP3,TUBA1A,TUBA1C,TUBB3,TUBB4A,VEGFA	9
Developmental Disorder,Hereditary Disorder,Organismal Injury and Abnormalities,Skeletal and Muscu	Autosomal dominant Emery-Dreifuss muscular dy	1,31E-03			ASPN,BGN,CELF2,COL6A1,COL6A2,CYB561,LOXL1,LTBP3,PLOD2,RASSF1,SDC2,SDCBP,SYNE2,TMEM43	14
Neurological Disease,Organismal Injury and Abnormalities,Psychological Disorders	Hypomyelinating leukodystrophy	1,31E-03			AIFM1,DEGS1,HIKESHI,POLR1C,POLR3B,TMEM106B,TMEM63A,TUBB4A	8
Cardiovascular Disease,Dermatological Diseases and Conditions,Hereditary Disorder,Organismal Injur	Hereditary hemorrhagic telangiectasia	1,32E-03			ACVRL1,BMPR2,SMAD4,VEGFA	4
Cancer,Organismal Injury and Abnormalities	Invasive adenocarcinoma	1,33E-03			AKT1,BCCIP,BCL2L1,BGN,BRCA2,CCND3,CD274,CHD1,ELP3,HSPB8,LGALS1,LTBP4,MAP2K2,MKI67,MYC,	32
Cellular Development	Epithelial-mesenchymal transition	1,35E-03		-0,352	AKT1,ARHGAP21,BTBD7,CAV1,CDKN1B,CLIC4,CSNK2B,CUL4A,DDX5,EIF3E,FBLN5,FLNA,FOXC2,FOXO3,I	44
Skeletal and Muscular Disorders	Muscle laminopathy	1,38E-03			ABCC9,ASPN,BAG3,BGN,CELF2,COL6A1,COL6A2,CYB561,EMD,LOXL1,LTBP3,PLOD2,RASSF1,SDC2,SDCB	18
Cell Cycle	G2/M phase	1,40E-03		-1,195	ARPP19,ATF5,BCL2L1,Calm1 (includes others),CAT,CCP110,CDKN1B,CELF2,CENPJ,CEP135,CEP152,CEP:	54
Cancer,Endocrine System Disorders,Organismal Injury and Abnormalities	Benign neoplasm of adrenal gland	1,45E-03			ALDH5A1,ASPN,BGN,COL6A1,COL6A2,DAD1,DDOST,EFEMP2,FLNC,GOLGA1,HRAS,MGAT1,OSTC,PRELP	19
Cancer,Organismal Injury and Abnormalities,Reproductive System Disease	Unresectable breast cancer	1,47E-03			AKT1,CD274,FKBP1A,PARP2,PARP3,TUBA1A,TUBA1C,TUBB3,TUBB4A,VEGFA	10
Cancer,Organismal Injury and Abnormalities	Metastasis of colorectal cancer cell lines	1,47E-03		-1,414	AKT1,CAV1,FOXC2,IRF1,KDM3A,MTDH,RBPJ,RICTOR,SH3BGRL,STIM1	10
Skeletal and Muscular Disorders	Laminopathy	1,49E-03			ABCC9,ASPN,BAG3,BGN,CELF2,COL6A1,COL6A2,CYB561,EMD,LOXL1,LTBP3,PLOD2,RASSF1,SDC2,SDCB	20
Cancer,Organismal Injury and Abnormalities,Reproductive System Disease	Recurrent HER2 negative invasive breast cancer	1,59E-03			CD274,CHD1,FKBP1A,PARP2,PARP3,TUBA1A,TUBA1C,TUBB,TUBB3,TUBB4A,VEGFA	11
Cancer,Endocrine System Disorders,Organismal Injury and Abnormalities,Reproductive System Diseas	Stage IV HER2 negative invasive breast cancer	1,59E-03			CD274,CHD1,FKBP1A,PARP2,PARP3,TUBA1A,TUBA1C,TUBB,TUBB3,TUBB4A,VEGFA	11
Cellular Development,Cellular Growth and Proliferation,Connective Tissue Development and Function	Differentiation of erythroid precursor cells	1,70E-03		-1,146	AXL,BCL2L1,BCR,BRD1,Cdc42,FLI1,HRAS,HSPA9,IRF1,MAPK1,MYC,RPS14,RPS19,RXRA,STAT5A,TET2,TIN	20
Cancer,Hematological Disease,Immunological Disease,Organismal Injury and Abnormalities	B-cell prolymphocytic leukemia	1,72E-03			BCLAF1,CHD2,MYC,SETD2,SF3B1	5
Developmental Disorder,Neurological Disease,Organismal Injury and Abnormalities	Dandy-Walker syndrome	1,72E-03			DPYSL5,PIBF1,PPP1CB,SETD2,TUBA1A	5
Cancer,Organismal Injury and Abnormalities,Reproductive System Disease	Nonmetastatic primary hormone receptor negati	1,80E-03			CD274,CHD1,PARP2,PARP3,TUBA1A,TUBA1C,TUBB3,TUBB4A	8
Cancer,Organismal Injury and Abnormalities	Relapsed primary peritoneal cancer	1,80E-03			CD274,PARP2,PARP3,TUBA1A,TUBA1C,TUBB3,TUBB4A,VEGFA	8
Cancer,Endocrine System Disorders,Organismal Injury and Abnormalities,Reproductive System Diseas	Non-mucinous ovarian carcinoma	1,80E-03			CD274,PARP2,PARP3,TUBA1A,TUBA1C,TUBB3,TUBB4A,VEGFA	8
Cancer,Endocrine System Disorders,Organismal Injury and Abnormalities,Reproductive System Diseas	BRCA2 mutation negative epithelial ovarian cance	1,80E-03			CD274,PARP2,PARP3,TUBA1A,TUBA1C,TUBB3,TUBB4A,VEGFA	8
Cancer,Organismal Injury and Abnormalities,Reproductive System Disease	Relapsed fallopian tube cancer	1,80E-03			CD274,PARP2,PARP3,TUBA1A,TUBA1C,TUBB3,TUBB4A,VEGFA	8
Cancer,Organismal Injury and Abnormalities,Tumor Morphology	Progressive primary peritoneal cancer	1,80E-03			CD274,PARP2,PARP3,TUBA1A,TUBA1C,TUBB3,TUBB4A,VEGFA	8
Cancer,Endocrine System Disorders,Organismal Injury and Abnormalities,Reproductive System Diseas	BRCA1 mutation negative epithelial ovarian cance	1,80E-03			CD274,PARP2,PARP3,TUBA1A,TUBA1C,TUBB3,TUBB4A,VEGFA	8
Cancer	Transformation of skin cell lines	1,80E-03		-1,528	HRAS,MAP3K8,MAPK1,MYC,RELA,SDCBP,SEM1	7
Cancer,Organismal Injury and Abnormalities	BRCA1 mutation negative BRCA2 mutation negati	1,80E-03			PARP2,PARP3,TUBA1A,TUBA1C,TUBB3,TUBB4A,VEGFA	7
Cancer,Organismal Injury and Abnormalities,Reproductive System Disease	BRCA1 mutation negative BRCA2 mutation negati	1,80E-03			PARP2,PARP3,TUBA1A,TUBA1C,TUBB3,TUBB4A,VEGFA	7
Cell-To-Cell Signaling and Interaction,Cellular Assembly and Organization	Quantity of focal adhesions	1,87E-03		0,293	ARHGAP35,CAPNS1,CAT,CAV1,CDH5,CFL2,FLNA,MYC,PTPN12,RAC1,RACK1,STRIP1,TIMP1,VEGFA	14
Cancer,Organismal Injury and Abnormalities,Reproductive System Disease	HER2 negative hormone receptor positive breast	1,88E-03			AKT1,CHD1,FKBP1A,PARP2,PARP3,TUBA1A,TUBA1C,TUBB3,TUBB4A,VEGFA	10
Connective Tissue Development and Function,Skeletal and Muscular System Development and Functi	Attachment of sternum	1,88E-03			BMPR2,Cdkn1c,CHUK,DEAF1,GNAI2,GNAI3,LMO4,SF3B1,SLC29A1,WFIKKN2	10
Cell Death and Survival	Cell death of fibrosarcoma cell lines	1,91E-03		1,475	BCL2L1,BMX,FTH1,LIMS1,NFKBIZ,OAZ1,RABL6,STAT1,STAT2,TBK1,TNFRSF10A,TNFSF10,TNFSF12,TRAF	16
Cell Death and Survival	Cell death of hybrid cells	1,98E-03		-0,736	AKT1,BCL2L1,DNAJA3,HRAS,LAMP2,MYC,OPTN,RELA,RORC,SIVA1,TNFAIP3	11
Cancer,Organismal Injury and Abnormalities,Reproductive System Disease	HER2 negative breast adenocarcinoma	1,98E-03			AKT1,CD274,CHD1,FKBP1A,PARP2,PARP3,TUBA1A,TUBA1C,TUBB3,TUBB4A,VEGFA	11
Gene Expression	Transcription of RNA	2,04E-03		0,86	ACTR3,ACVRL1,AFAP1L2,AGO1,AGO2,AKAP8L,AKT1,ARF4,ASXL2,ATAD2,ATF5,ATN1,ATP2B4,BAD,BCL2	278
Developmental Disorder,Hereditary Disorder,Organismal Injury and Abnormalities,Skeletal and Muscu	Dystrophy of muscle	2,07E-03			ACE,ADRA1A,ASPN,BGN,CCND3,CELF2,CNBP,COL6A1,COL6A2,CYB561,DAG1,DDX5,DPAGT1,DPM3,EM	43
Cancer,Organismal Injury and Abnormalities,Reproductive System Disease	Triple negative breast adenocarcinoma	2,17E-03			AKT1,CD274,CHD1,PARP2,PARP3,TUBA1A,TUBA1C,TUBB3,TUBB4A	9
Cancer,Endocrine System Disorders,Organismal Injury and Abnormalities,Reproductive System Diseas	Advanced HER2 negative hormone receptor nega	2,17E-03			AKT1,CD274,CHD1,TUBA1A,TUBA1C,TUBB,TUBB3,TUBB4A,VEGFA	9
Cancer,Endocrine System Disorders,Organismal Injury and Abnormalities	Adrenal gland tumor	2,28E-03			ACVRL1,ADRA1A,ALDH5A1,ASPN,BGN,BMPR2,CDKN1B,COL6A1,COL6A2,DAD1,DDOST,EFEMP2,FLNC,(	32
Cancer,Endocrine System Disorders,Organismal Injury and Abnormalities,Reproductive System Diseas	Stage IV estrogen receptor positive HER2 negativ	2,37E-03			CHD1,FKBP1A,PARP2,PARP3,TUBA1A,TUBA1C,TUBB,TUBB3,TUBB4A,VEGFA	10
Cancer,Endocrine System Disorders,Organismal Injury and Abnormalities,Reproductive System Diseas	Stage IV estrogen receptor positive HER2 negativ	2,37E-03			CHD1,FKBP1A,PARP2,PARP3,TUBA1A,TUBA1C,TUBB,TUBB3,TUBB4A,VEGFA	10

Categories	Diseases or Functions Annotation	p-value	Predicted Activation State	Activation z-score	Molecules	# Molecules
Cancer,Organismal Injury and Abnormalities,Respiratory Disease	ALK fusion negative EGFR mutation negative non:	2,37E-03			ATIC,AXL,CD274,PARP2,PARP3,TUBA1A,TUBA1C,TUBB3,TUBB4A,VEGFA	10
Cancer,Organismal Injury and Abnormalities,Reproductive System Disease	Recurrent estrogen receptor positive HER2 negat	2,37E-03			CHD1,FKBP1A,PARP2,PARP3,TUBA1A,TUBA1C,TUBB,TUBB3,TUBB4A,VEGFA	10
Cancer,Endocrine System Disorders,Organismal Injury and Abnormalities,Reproductive System Diseas	Stage IV HER2 negative progesterone receptor pc	2,37E-03			CHD1,FKBP1A,PARP2,PARP3,TUBA1A,TUBA1C,TUBB,TUBB3,TUBB4A,VEGFA	10
Cancer,Organismal Injury and Abnormalities	Metastasis of adenocarcinoma	2,38E-03		-0,57	AKT1,CBR1,LGALS1,MYC,THRA,THRB	6
Cancer,Organismal Injury and Abnormalities	Primary peritoneal mixed carcinoma	2,41E-03			CD274,PARP2,PARP3,TUBA1A,TUBA1C,TUBB3,TUBB4A,VEGFA	8
Cancer,Organismal Injury and Abnormalities,Reproductive System Disease	Recurrent uterine sarcoma	2,41E-03			CD274,PARP2,PARP3,TUBA1A,TUBA1C,TUBB3,TUBB4A,VEGFA	8
Cancer,Organismal Injury and Abnormalities	Primary BRCA2 mutation positive peritoneal canc	2,41E-03			CD274,PARP2,PARP3,TUBA1A,TUBA1C,TUBB3,TUBB4A,VEGFA	8
Cancer,Organismal Injury and Abnormalities	Primary recurrent peritoneal cancer	2,41E-03			CD274,PARP2,PARP3,TUBA1A,TUBA1C,TUBB3,TUBB4A,VEGFA	8
Cancer,Organismal Injury and Abnormalities	BRCA1 mutation positive primary peritoneal canc	2,41E-03			CD274,PARP2,PARP3,TUBA1A,TUBA1C,TUBB3,TUBB4A,VEGFA	8
Cancer,Organismal Injury and Abnormalities	BRCA2 mutation negative peritoneal cancer	2,41E-03			CD274,PARP2,PARP3,TUBA1A,TUBA1C,TUBB3,TUBB4A,VEGFA	8
Cancer,Organismal Injury and Abnormalities	BRCA1 mutation negative peritoneal cancer	2,41E-03			CD274,PARP2,PARP3,TUBA1A,TUBA1C,TUBB3,TUBB4A,VEGFA	8
Cancer,Immunological Disease,Organismal Injury and Abnormalities	Thymoma	2,44E-03			AKT1,CASP1,ERCC1,FKBP1A,HRAS,MKI67,MYC,TUBA1A,TUBA1C,TUBB3,TUBB4A	11
Organismal Development	Growth of multicellular organism	2,47E-03			ATF5,ATN1,BRCA2,Cdkn1c,CELA1,CLIC4,ERCC1,ERCC2,ERCC5,ERCC6,H3-3A/H3-3B,HLA-A,KDM2A,NEK1	21
Infectious Diseases	Viral Infection	2,49E-03	Decreased	-5,416	ABCD2,ACE,ACE2,ACTR3,ADRA1A,AFG3L2,AGO1,AGO2,AHSG,AKT1,ALKBH3,ANKRD6,ANXA5,AP2A1,AF	274
Skeletal and Muscular System Development and Function	Morphology of muscle	2,50E-03			ACADM,ACE,ACVRL1,ADAM12,ADRA1A,AKT1,BAG3,BCL2L1,BGN,BMX,CAPN6,CAPNS1,CAT,CAV1,CD55	95
Cancer,Hereditary Disorder,Organismal Injury and Abnormalities	Hereditary cancer-predisposing syndrome	2,52E-03			AKT1,AXIN2,AXL,BCL2L1,BRCA2,CAT,CD274,CDKN1B,CHIC2,CYB561,DDX41,EHBP1,EIF2AK4,EPHB2,ERC	70
Cancer,Organismal Injury and Abnormalities,Reproductive System Disease	Stage IV fallopian tube cancer	2,53E-03			PARP2,PARP3,TUBA1A,TUBA1C,TUBB3,TUBB4A,VEGFA	7
Cancer,Organismal Injury and Abnormalities,Respiratory Disease,Tumor Morphology	Stage IV progressive EGFR activating mutation po	2,53E-03			ATIC,CD274,TUBA1A,TUBA1C,TUBB3,TUBB4A,VEGFA	7
Cancer,Organismal Injury and Abnormalities,Reproductive System Disease	Unresectable HER2 negative breast adenocarcino	2,53E-03			AKT1,CD274,TUBA1A,TUBA1C,TUBB3,TUBB4A,VEGFA	7
Cancer,Organismal Injury and Abnormalities,Respiratory Disease	Stage IV ALK translocation positive nonsquamous	2,53E-03			ATIC,CD274,TUBA1A,TUBA1C,TUBB3,TUBB4A,VEGFA	7
Cancer,Organismal Injury and Abnormalities	Stage III-IV primary peritoneal carcinoma	2,53E-03			CD274,PARP2,TUBA1A,TUBA1C,TUBB3,TUBB4A,VEGFA	7
Cell Cycle	G2 phase	2,66E-03		-0,99	AKT1,ARPP19,ATF5,BCL2L1,Calm1 (includes others),CAT,CCP110,CDKN1B,CELF2,CENPJ,CEP135,CEP15	61
Embryonic Development,Organismal Development,Tissue Morphology	Morphology of germ layer	2,78E-03			BMPR2,CR1L,DAD1,DAG1,DHX9,DRAP1,IL11RA,MAPK1,PBX1,PIKFYVE,PPP2CA,PTGIS,RAB10,RAC1,SMA	21
Cancer,Organismal Injury and Abnormalities,Reproductive System Disease	Advanced hormone receptor positive breast canc	2,78E-03			AKT1,CD274,CHD1,FKBP1A,HDAC10,PARP2,PARP3,TUBA1A,TUBA1C,TUBB,TUBB3,TUBB4A,VEGFA	13
Cardiovascular Disease,Hematological Disease,Organismal Injury and Abnormalities	Aplastic anemia	2,79E-03			BRCA2,CAT,DDX41,FKBP1A,GPX1,PPP3R1,RPL18,RPL26,RPL35,RPL35A,RPS10,RPS15A,RPS19,RPS20,RP	19
Cancer,Organismal Injury and Abnormalities,Respiratory Disease	Early stage non-small cell lung cancer	2,82E-03			ACTR10,AGO1,AGO2,ATIC,CD274,EEF1A1,RHOC,RPL7,RPS11,RPS27A,SSR2,TUBA1A,TUBA1C,TUBB3,TU	15
Cancer,Organismal Injury and Abnormalities	Peritoneal cavity cancer	2,82E-03			ANGPT1,ATIC,BRCA2,CD274,CHD1,IFNGR1,MAP2K2,MAPK1,PARP2,PARP3,TUBA1A,TUBA1C,TUBB3,TU	15
Cell Morphology,Hematological System Development and Function	Morphology of B-lymphocyte derived cell lines	2,84E-03			AKT1,BCL2L1,CRKL,MYC	4
Cellular Assembly and Organization	Dispersal of Golgi apparatus	2,84E-03		-1,982	BRD8,GOLPH3,RASSF1,RINT1	4
Cancer,Dermatological Diseases and Conditions,Organismal Injury and Abnormalities	Hyperplasia of keratinocytes	2,84E-03		0,152	RAC1,RXRA,THRA,THRB	4
Cardiovascular System Development and Function,Hematological System Development and Function	Diastolic pressure of left ventricle	2,84E-03		1,067	ACE2,HSPB8,ITGB3,TIMP1	4
Amino Acid Metabolism,Small Molecule Biochemistry	Synthesis of glutamine	2,84E-03		1,154	IDH1,IKBKE,MYC,PRKAR1A	4
DNA Replication, Recombination, and Repair	Metabolism of DNA	2,84E-03		0,735	AAGAB,ACIN1,ACVRL1,AIFM1,AKAP8L,AKT1,ANGPT1,ANTXR1,APOBEC3B,BANF1,BCL2L1,BMX,BRCA2,C	69
Cardiovascular Disease,Hereditary Disorder,Organismal Injury and Abnormalities,Respiratory Disease	Familial primary pulmonary hypertension	2,89E-03			ACVRL1,BMPR2,CAV1,EIF2AK4,SMAD4	5
Developmental Disorder,Hereditary Disorder,Metabolic Disease,Organismal Injury and Abnormalities	Acyl-coa dehydrogenase deficiency	2,89E-03			ACAD8,ACAD9,ACADL,ACADM,ETFDH	5
Cancer,Organismal Injury and Abnormalities,Reproductive System Disease	Fallopian tube serous adenocarcinoma	2,95E-03			CD274,MAP2K2,MAPK1,PARP2,PARP3,TUBA1A,TUBA1C,TUBB3,TUBB4A,VEGFA	10
Cancer,Hematological Disease,Hereditary Disorder,Organismal Injury and Abnormalities	Constitutional bone marrow failure syndrome	2,98E-03			BRCA2,CAT,FKBP1A,GPX1,RPL18,RPL26,RPL35,RPL35A,RPS10,RPS15A,RPS19,RPS20,RPS24,RPS26,RPS2	16
Cancer,Organismal Injury and Abnormalities	Progesterone receptor positive cancer	2,98E-03			AKT1,CHD1,FKBP1A,GON4L,HSPB8,LTBP3,PARP2,PARP3,PLK2,SF3B1,TUBA1A,TUBA1C,TUBB,TUBB3,TU	16
Infectious Diseases,Organismal Injury and Abnormalities	Infection of embryonic cell lines	3,10E-03	Decreased	-2,555	ACE2,AFG3L2,ALKBH3,AXL,CD164,CLIP3,DAG1,DAZAP2,DBN1,DDX23,DPYSL5,ERCC1,ERCC5,ERI3,FAM1	48
Hematological Disease	Failure of bone marrow	3,14E-03			BRCA2,CAT,FKBP1A,GPX1,IFNAR1,MDM4,RPL18,RPL26,RPL35,RPL35A,RPS10,RPS15A,RPS19,RPS20,RP	18
Cell Morphology,Connective Tissue Development and Function	Morphology of fibroblast cell lines	3,15E-03			ACVRL1,ADAM12,AFAP1L1,AKT1,BCL2L1,CAV1,Cdc42,CDC42EP5,CDKN1B,DPY30,ECM1,HRAS,KRT18,M	20
Cell Signaling	I-kappaB kinase/NF-kappaB cascade	3,16E-03		-1,266	AKT1,ANGPT1,CASP1,CHUK,ECM1,EEF1D,FKBP1A,FLNA,IRF1,LGALS1,MALT1,MAP3K14,MTDH,NDFIP1,	35
Cancer,Inflammatory Disease,Inflammatory Response,Organismal Injury and Abnormalities,Reproduc	Nonmetastatic inflammatory breast cancer	3,18E-03			CD274,CHD1,PARP2,PARP3,TUBA1A,TUBA1C,TUBB3,TUBB4A	8
Cancer,Organismal Injury and Abnormalities	High grade primary peritoneal serous adenocarci	3,18E-03			CD274,PARP2,PARP3,TUBA1A,TUBA1C,TUBB3,TUBB4A,VEGFA	8
Cancer,Organismal Injury and Abnormalities,Reproductive System Disease	High grade fallopian tube endometrioid adenocai	3,18E-03			CD274,PARP2,PARP3,TUBA1A,TUBA1C,TUBB3,TUBB4A,VEGFA	8
Cancer,Endocrine System Disorders,Organismal Injury and Abnormalities,Reproductive System Diseas	BRCA2 mutation positive ovarian carcinoma	3,18E-03			CD274,PARP2,PARP3,TUBA1A,TUBA1C,TUBB3,TUBB4A,VEGFA	8
Cancer,Endocrine System Disorders,Organismal Injury and Abnormalities,Reproductive System Diseas	BRCA1 mutation positive ovarian carcinoma	3,18E-03			CD274,PARP2,PARP3,TUBA1A,TUBA1C,TUBB3,TUBB4A,VEGFA	8
Cancer,Organismal Injury and Abnormalities,Reproductive System Disease	BRCA1 mutation negative fallopian tube cancer	3,18E-03			CD274,PARP2,PARP3,TUBA1A,TUBA1C,TUBB3,TUBB4A,VEGFA	8
Cancer,Organismal Injury and Abnormalities,Reproductive System Disease	BRCA1 mutation positive fallopian tube cancer	3,18E-03			CD274,PARP2,PARP3,TUBA1A,TUBA1C,TUBB3,TUBB4A,VEGFA	8
Cancer,Organismal Injury and Abnormalities,Reproductive System Disease	BRCA2 mutation negative fallopian tube cancer	3,18E-03			CD274,PARP2,PARP3,TUBA1A,TUBA1C,TUBB3,TUBB4A,VEGFA	8
Cancer,Organismal Injury and Abnormalities	High grade primary peritoneal endometrioid carc	3,18E-03			CD274,PARP2,PARP3,TUBA1A,TUBA1C,TUBB3,TUBB4A,VEGFA	8
Cancer,Organismal Injury and Abnormalities,Reproductive System Disease	BRCA2 mutation positive fallopian tube cancer	3,18E-03			CD274,PARP2,PARP3,TUBA1A,TUBA1C,TUBB3,TUBB4A,VEGFA	8
Cancer,Endocrine System Disorders,Organismal Injury and Abnormalities,Reproductive System Diseas	High grade ovarian endometrioid carcinoma	3,18E-03			CD274,PARP2,PARP3,TUBA1A,TUBA1C,TUBB3,TUBB4A,VEGFA	8
Cancer,Organismal Injury and Abnormalities,Reproductive System Disease	Primary triple negative breast cancer	3,18E-03			CD274,CHD1,PARP2,PARP3,TUBA1A,TUBA1C,TUBB3,TUBB4A	8
Cancer,Organismal Injury and Abnormalities,Reproductive System Disease	Advanced fallopian tube cancer	3,18E-03			CD274,PARP2,PARP3,TUBA1A,TUBA1C,TUBB3,TUBB4A,VEGFA	8
Cell Cycle	Mitosis	3,20E-03		-1,508	AFAP1L2,AHSG,AKAP8,AKT1,ANAPC11,ARPP19,AXL,BCCIP,BRAP,BRCA2,BRD2,BUB3,CCP110,CCT4,Cdc4	89
Cell Death and Survival,Organismal Injury and Abnormalities	Necrosis of vascular endothelial cells	3,23E-03		0,513	ACVRL1,AKT1,ANGPT1,ARHGAP18,ATP1B3,AXL,BCL2L1,CASP1,CAT,CAV1,CD274,EFEMP2,FOXO3,LGAL	24
Cancer,Organismal Injury and Abnormalities,Reproductive System Disease	Metastatic HER2-negative breast cancer	3,29E-03			AKT1,BRCA2,CD274,CHD1,FKBP1A,PARP2,PARP3,STAT1,TUBA1A,TUBA1C,TUBB3,TUBB4A,VEGFA	13
Hereditary Disorder,Organismal Injury and Abnormalities,Skeletal and Muscular Disorders	Autosomal recessive myopathy	3,35E-03			AP4S1,CFL2,COL6A1,COL6A2,COX15,CYP1B1,DAG1,DDHD1,DDHD2,DPAGT1,DPM3,GBA2,HADHA,HAD	41
Cardiovascular Disease,Hematological Disease,Organismal Injury and Abnormalities	Anemia	3,39E-03	Increased	2,008	AGO2,AKT1,AXL,BCL2L1,BRCA2,BRD1,CAT,CCND3,Cdc42,DAD1,DDX41,DFFB,FKBP1A,FLI1,FOXO3,GIM	80
Cell Death and Survival,Organismal Injury and Abnormalities	Apoptosis of vascular endothelial cells	3,41E-03		0,363	ACVRL1,AKT1,ANGPT1,ARHGAP18,ATP1B3,AXL,BCL2L1,CASP1,CAT,CAV1,CD274,EFEMP2,FOXO3,LGAL	22
Cancer,Organismal Injury and Abnormalities,Reproductive System Disease	Recurrent endometrial carcinosarcoma	3,47E-03			CD274,PARP2,PARP3,TUBA1A,TUBA1C,TUBB3,TUBB4A	7
Cancer,Organismal Injury and Abnormalities,Reproductive System Disease	Primary HER2 negative invasive breast carcinoma	3,47E-03			CD274,PARP2,PARP3,TUBA1A,TUBA1C,TUBB3,TUBB4A	7
Cancer,Organismal Injury and Abnormalities,Respiratory Disease	ALK rearrangement positive CD274 positive non-s	3,47E-03			ATIC,CD274,TUBA1A,TUBA1C,TUBB3,TUBB4A,VEGFA	7
Cancer,Organismal Injury and Abnormalities,Respiratory Disease	Stage IV CD274 low expression positive non-squa	3,47E-03			ATIC,CD274,TUBA1A,TUBA1C,TUBB3,TUBB4A,VEGFA	7
Cancer,Hereditary Disorder,Organismal Injury and Abnormalities,Reproductive System Disease	Triple-negative familial breast cancer	3,47E-03			AKT1,PARP2,PARP3,TUBA1A,TUBA1C,TUBB3,TUBB4A	7

Categories	Diseases or Functions Annotation	p-value	Predicted Activation State	Activation z-score	Molecules	# Molecules
Cancer,Organismal Injury and Abnormalities,Respiratory Disease	Stage IV CD274 overexpression positive non-squa	3,47E-03			ATIC,CD274,TUBA1A,TUBA1C,TUBB3,TUBB4A,VEGFA	7
Cancer,Endocrine System Disorders,Organismal Injury and Abnormalities,Reproductive System Diseas	Locally advanced triple negative breast adenocar	3,48E-03			AKT1,CD274,TUBA1A,TUBA1C,TUBB3,TUBB4A	6
Cancer,Organismal Injury and Abnormalities,Reproductive System Disease	Unresectable triple negative breast adenocarcino	3,48E-03			AKT1,CD274,TUBA1A,TUBA1C,TUBB3,TUBB4A	6
Cell Cycle,Embryonic Development	Arrest in G2/M phase of embryonic cell lines	3,48E-03			CDKN1B,OGT,PPP2CA,PPP2CB,RASSF1,RBBP6	6
Cancer,Organismal Injury and Abnormalities	Primary advanced cancer	3,53E-03			CD274,CHD1,PARP2,PARP3,TUBA1A,TUBA1C,TUBB3,TUBB4A,VEGFA	9
Cancer,Organismal Injury and Abnormalities	Primary peritoneal cancer	3,58E-03			ANGPT1,ATIC,BRCA2,CD274,CHD1,MAP2K2,MAPK1,PARP2,PARP3,TUBA1A,TUBA1C,TUBB3,TUBB4A,VE	14
Cancer,Endocrine System Disorders,Organismal Injury and Abnormalities,Reproductive System Diseas	Recurrent ovarian cancer	3,62E-03			BRCA2,CD274,MAP2K2,MAPK1,PARP2,PARP3,TUBA1A,TUBA1C,TUBB3,TUBB4A,VEGFA	11
Cancer,Organismal Injury and Abnormalities,Reproductive System Disease	Metastatic HER2 negative hormone receptor neg	3,62E-03			AKT1,BRCA2,CD274,CHD1,PARP2,PARP3,STAT1,TUBA1A,TUBA1C,TUBB3,TUBB4A	11
Cancer,Organismal Injury and Abnormalities,Reproductive System Disease	Stage IV hormone receptor positive advanced bre	3,65E-03			CHD1,FKBP1A,PARP2,PARP3,TUBA1A,TUBA1C,TUBB,TUBB3,TUBB4A,VEGFA	10
Cancer,Organismal Injury and Abnormalities,Reproductive System Disease	Invasive estrogen receptor positive progesterone	3,65E-03			CHD1,FKBP1A,PARP2,PARP3,TUBA1A,TUBA1C,TUBB,TUBB3,TUBB4A,VEGFA	10
Cancer,Inflammatory Disease,Inflammatory Response,Organismal Injury and Abnormalities,Reproduc	Inflammatory breast cancer	3,65E-03			CD274,CHD1,FKBP1A,NOTCH3,PARP2,PARP3,TUBA1A,TUBA1C,TUBB3,TUBB4A	10
Cancer,Organismal Injury and Abnormalities,Reproductive System Disease	Recurrent estrogen receptor positive progesteror	3,65E-03			CHD1,FKBP1A,PARP2,PARP3,TUBA1A,TUBA1C,TUBB,TUBB3,TUBB4A,VEGFA	10
Developmental Disorder,Hereditary Disorder,Organismal Injury and Abnormalities,Skeletal and Muscu	Progressive muscular dystrophy	3,81E-03			ACE,ADRA1A,ASPN,BGN,CELF2,COL6A1,COL6A2,CYB561,DAG1,DPAGT1,DPM3,HLA-A,HNRNPDL,LOXL1	27
Cellular Compromise	Breakage of sister chromatids	3,82E-03			CDKN1B,PARP2,UIMC1	3
Cell Cycle	Arrest in M phase of kidney cell lines	3,82E-03			FBXO5,NEK9,RASSF1	3
Hematological System Development and Function,Lymphoid Tissue Structure and Development,Tissu	Quantity of natural T-regulatory cells	3,82E-03			RICTOR,TGFBR1,TSC1	3
Neurological Disease	Degeneration of dorsal root ganglion	3,82E-03			ABCD2,AKT1,VTI1B	3
Cellular Development,Cellular Growth and Proliferation	Expansion of pheochromocytoma cell lines	3,82E-03			AKT1,HRAS,RAC1	3
Cellular Movement	Invasion by Salmonella typhimurium SL1344	3,82E-03			Cdc42,NOD2,RAC1	3
Cell-To-Cell Signaling and Interaction	Detachment of fibroblast cell lines	3,82E-03			FLNA,MAPK1,MYC	3
Developmental Disorder,Hereditary Disorder,Organismal Injury and Abnormalities,Skeletal and Muscu	Myotonic dystrophy type 2	3,82E-03			CCND3,CNBP,DDX5	3
Cell Death and Survival,Cell Morphology,Cellular Function and Maintenance	Permeability of lung cell lines	3,82E-03			ANGPT1,ARHGAP35,RAC1	3
Cancer,Organismal Injury and Abnormalities	Stage II-IV BRCA1 mutation negative BRCA2 muta	3,82E-03			PARP2,PARP3,VEGFA	3
Cancer,Endocrine System Disorders,Hereditary Disorder,Organismal Injury and Abnormalities,Reprod	Stage II-IV BRCA1 germline mutation positive epit	3,82E-03			PARP2,PARP3,VEGFA	3
Cancer,Endocrine System Disorders,Hereditary Disorder,Organismal Injury and Abnormalities,Reprod	Stage II-IV BRCA2 germline mutation positive epit	3,82E-03			PARP2,PARP3,VEGFA	3
Cancer,Hereditary Disorder,Organismal Injury and Abnormalities	Stage II-IV primary BRCA1 germline mutation pos	3,82E-03			PARP2,PARP3,VEGFA	3
Cancer,Hereditary Disorder,Organismal Injury and Abnormalities,Reproductive System Disease	Stage II-IV BRCA2 germline mutation positive fallc	3,82E-03			PARP2,PARP3,VEGFA	3
Cancer,Organismal Injury and Abnormalities	BRCA rearrangement positive peritoneal cancer	3,82E-03			PARP2,PARP3,VEGFA	3
Cancer,Organismal Injury and Abnormalities,Reproductive System Disease	Stage II-IV BRCA2 somatic mutation positive fallo	3,82E-03			PARP2,PARP3,VEGFA	3
Cancer,Organismal Injury and Abnormalities	Stage II-IV BRCA2 somatic mutation positive perit	3,82E-03			PARP2,PARP3,VEGFA	3
Cancer,Organismal Injury and Abnormalities,Reproductive System Disease	BRCA rearrangement positive fallopian tube canc	3,82E-03			PARP2,PARP3,VEGFA	3
Cancer,Hereditary Disorder,Organismal Injury and Abnormalities,Reproductive System Disease	Stage II-IV BRCA1 germline mutation positive fallc	3,82E-03			PARP2,PARP3,VEGFA	3
Cancer,Hereditary Disorder,Organismal Injury and Abnormalities	Stage II-IV primary BRCA2 germline mutation pos	3,82E-03			PARP2,PARP3,VEGFA	3
Cancer,Endocrine System Disorders,Organismal Injury and Abnormalities,Reproductive System Diseas	Stage II-IV BRCA1 somatic mutation positive epith	3,82E-03			PARP2,PARP3,VEGFA	3
Lipid Metabolism,Molecular Transport,Small Molecule Biochemistry	Uptake of lauric acid	3,82E-03			CAV1,EHD2,SLC27A4	3
Cancer,Organismal Injury and Abnormalities	Stage II-IV BRCA1 somatic mutation positive perit	3,82E-03			PARP2,PARP3,VEGFA	3
Cancer,Organismal Injury and Abnormalities,Reproductive System Disease	Stage II-IV BRCA1 somatic mutation positive fallo	3,82E-03			PARP2,PARP3,VEGFA	3
Cancer,Endocrine System Disorders,Organismal Injury and Abnormalities,Reproductive System Diseas	Stage II-IV BRCA2 somatic mutation positive epith	3,82E-03			PARP2,PARP3,VEGFA	3
Cancer,Endocrine System Disorders,Organismal Injury and Abnormalities,Reproductive System Diseas	BRCA rearrangement positive ovarian cancer	3,82E-03			PARP2,PARP3,VEGFA	3
Cellular Movement,Hematological System Development and Function,Immune Cell Trafficking,Inflamr	Migration of dermal dendritic cells	3,82E-03			Cdc42,LGALS1,RAMP1	3
Infectious Diseases	Replication of Myxoma virus	3,82E-03			DHX9,IFNAR1,STAT1	3
Hematological System Development and Function,Immunological Disease,Lymphoid Tissue Structure	Abnormal morphology of enlarged submandibula	3,82E-03			ATP2B4,TOP3B,TRAF3IP2	3
Cell Cycle	Interphase	3,92E-03		-0,338	ACIN1,ACVRL1,ADRA1A,AFAP1L2,AKT1,ANGPT1,Anp32b,ARPP19,ATF5,AXL,BAD,BAG3,BCL2L1,Calm1 (	124
Cancer,Endocrine System Disorders,Organismal Injury and Abnormalities	Adrenal cortex adenoma	4,00E-03			ALDH5A1,ASPN,BGN,COL6A1,COL6A2,DAD1,DDOST,EFEMP2,FLNC,MGAT1,OSTC,PRELP,PRKAR1A,RHO	17
Cell Cycle,Cellular Development,Connective Tissue Development and Function	Senescence of fibroblast cell lines	4,11E-03		-0,925	AKT1,BCL2L12,BRD7,BUB3,CAV1,CIRBP,DPY30,Ewsr1,GPATCH8,HRAS,KDM2A,MAPK1,MORC3,MYC,NE	29
Cell Death and Survival	Apoptosis of vascular cells	4,11E-03		0,543	ACE2,ACVRL1,AKT1,ANGPT1,ARHGAP18,ATP1B3,AXL,BCL2L1,CASP1,CAT,CAV1,CD274,EEF1D,EFEMP2,	29
Cancer,Organismal Injury and Abnormalities,Reproductive System Disease	Recurrent breast carcinoma	4,13E-03			AKT1,CD274,FKBP1A,TUBA1A,TUBA1C,TUBB3,TUBB4A,VEGFA	8
Cancer,Endocrine System Disorders,Organismal Injury and Abnormalities,Reproductive System Diseas	Low-grade serous ovarian adenocarcinoma	4,13E-03			MAP2K2,MAPK1,PARP2,PARP3,TUBA1A,TUBA1C,TUBB3,TUBB4A	8
Cancer,Organismal Injury and Abnormalities,Reproductive System Disease	Primary HER2 negative invasive breast cancer	4,13E-03			CD274,CHD1,PARP2,PARP3,TUBA1A,TUBA1C,TUBB3,TUBB4A	8
Cancer,Organismal Injury and Abnormalities	Recurrent primary peritoneal cancer	4,13E-03			CD274,PARP2,PARP3,TUBA1A,TUBA1C,TUBB3,TUBB4A,VEGFA	8
Cancer,Organismal Injury and Abnormalities	Advanced primary peritoneal cancer	4,13E-03			CD274,PARP2,PARP3,TUBA1A,TUBA1C,TUBB3,TUBB4A,VEGFA	8
Gene Expression	Transcription of DNA	4,30E-03		-0,312	ACTR3,ACVRL1,AFAP1L2,AGO1,AGO2,AKAP8L,AKT1,ARF4,ASXL2,ATAD2,ATF5,ATN1,ATP2B4,BCL2L12,E	228
Organismal Injury and Abnormalities	Benign lesion	4,33E-03		0,031	ACE,ADAM12,ADRA1A,AIG1,AKAP8L,AKT1,ALDH5A1,ANAPC10,ANGPT1,ANTXR1,ANXA5,ARHGDIA,ASP	174
Cancer,Organismal Injury and Abnormalities,Reproductive System Disease	Advanced estrogen receptor positive breast canc	4,36E-03			CHD1,FKBP1A,HDAC10,PARP2,PARP3,TUBA1A,TUBA1C,TUBB,TUBB3,TUBB4A,VEGFA	11
Cardiovascular System Development and Function,Cell Morphology,Cellular Development,Organismal	Tubulation of endothelial cell lines	4,36E-03		0,602	CASP1,CAVIN2,CDH5,CLIC4,CXCL10,ORAI1,PRKACB,RAP1A,SETD2,STIM1,VEGFA	11
Cell Death and Survival,Gastrointestinal Disease,Hepatic System Disease,Organismal Injury and Abnor	Cell death of liver cell lines	4,47E-03		0,431	AKT1,BAD,BCL2L1,FOXO3,LAMP2,LAPTM4B,MYC,TIMP1,TNFSF10,TNFSF12	10
Cancer,Dermatological Diseases and Conditions,Organismal Injury and Abnormalities	Development of skin tumor	4,49E-03		0,543	AKT1,BRCA2,CASP1,CCND3,CLIC4,GSTM1,HRAS,IL17RC,OAZ1,RAC1,RHEB,SMO,SPINT2,TRAF3IP2,VEGF	16
Infectious Diseases	Infection of cells	4,53E-03	Decreased	-4,514	ACE2,ACTR3,AFG3L2,AKT1,ALKBH3,ANKRD6,AP2A1,APOBEC3B,ASXL2,ATG16L2,ATP6V0A1,ATP6V0D1,	126
Cell Death and Survival,Skeletal and Muscular Disorders	Apoptosis of smooth muscle cell lines	4,54E-03		1,387	IRF1,NOTCH3,RAC1,TNFRSF10A,TNFSF10	5
Cell Morphology	Polarization of leukemia cell lines	4,54E-03		0	AHSG,DPYSL2,IGSF8,RAC1,RICTOR	5
Tissue Development	Formation of elastic fibers	4,54E-03			EFEMP2,FBLN5,LOXL1,LTBP4,MFAP4	5
Cell Cycle	Exit from G1 phase	4,54E-03			CDKN1B,CSNK2B,MYC,RASSF1,RBM5	5
Cardiovascular System Development and Function,Cell Morphology,Organismal Development	Sprouting of capillary vessel	4,54E-03		0	ACVRL1,CAT,CD63,ITGB1BP1,VEGFA	5
Cancer,Organismal Injury and Abnormalities	Initiation of formation of tumor	4,54E-03			FOXC2,FTO,HRAS,MYC,SMO	5
Cancer,Organismal Injury and Abnormalities,Tissue Morphology	Quantity of tumor	4,65E-03		-0,291	AKT1,ARL6IP5,ATAD3A,BMPR2,BRCA2,CAV1,CD151,CDKN1B,Cdkn1c,CYP1B1,EIF4EBP2,FOXC2,FOXO1,	36
Cancer,Hereditary Disorder,Organismal Injury and Abnormalities	BRCA2 germline mutation positive cancer	4,65E-03			PARP2,PARP3,TUBA1A,TUBA1C,TUBB3,TUBB4A,VEGFA	7

Categories	Diseases or Functions Annotation	p-value	Predicted Activation State	Activation z-score	Molecules	# Molecules
Cancer,Hereditary Disorder,Organismal Injury and Abnormalities	BRCA1 germline mutation positive cancer	4,65E-03			PARP2,PARP3,TUBA1A,TUBA1C,TUBB3,TUBB4A,VEGFA	7
Cancer,Organismal Injury and Abnormalities	BRCA germline mutation negative cancer	4,65E-03			PARP2,PARP3,TUBA1A,TUBA1C,TUBB3,TUBB4A,VEGFA	7
Cell Death and Survival	Cell death of fibroblast cell lines	4,73E-03		-0,026	ADAM12,AIFM1,AKT1,ATN1,BAD,BCL2L1,BRCA2,C1orf159,CA3,CAPNS1,CDKN1B,Cdkn1c,CLIC4,DNAJA:	74
Cancer,Organismal Injury and Abnormalities,Reproductive System Disease	Invasive ductal breast carcinoma	4,78E-03			BCCIP,BCL2L1,BGN,BRCA2,CCND3,CD274,CHD1,ELP3,HSPB8,LGALS1,LTBP4,MKI67,PTPRF,RASSF1,RELA	24
Cell Death and Survival	Cell death of tumor cell lines	4,79E-03		1,597	ADRM1,AGO1,AIFM1,AKT1,AKT1S1,ALKBH3,ANXA5,ARHGDIA,ATAD2,ATF5,ATN1,ATP1B3,AVPI1,AXL,B.	252
Cancer,Organismal Injury and Abnormalities,Reproductive System Disease	Recurrent breast cancer	4,83E-03			AKT1,CD274,CHD1,FKBP1A,HDAC10,PARP2,PARP3,RRAS2,TUBA1A,TUBA1C,TUBB,TUBB3,TUBB4A,VEG	14
Cell Death and Survival	Cell death of lung cell lines	4,91E-03	Increased	2,182	AFAP1L2,AKT1,BCL2L1,CDKN1B,CLK2,HRAS,IFNAR1,MAP3K5,MYC,TNFAIP3,TNFSF10,TXN	12
Cancer,Endocrine System Disorders,Organismal Injury and Abnormalities,Reproductive System Diseas	Advanced stage HER2-negative breast cancer	4,91E-03			AKT1,CD274,CHD1,FKBP1A,PARP2,PARP3,TUBA1A,TUBA1C,TUBB,TUBB3,TUBB4A,VEGFA	12
Cancer,Organismal Injury and Abnormalities,Reproductive System Disease	Recurrent HER2-negative breast cancer	4,91E-03			AKT1,CD274,CHD1,FKBP1A,PARP2,PARP3,TUBA1A,TUBA1C,TUBB,TUBB3,TUBB4A,VEGFA	12
Cancer,Endocrine System Disorders,Organismal Injury and Abnormalities,Reproductive System Diseas	Stage III-IV high-grade serous ovarian carcinoma	4,92E-03			CD274,PARP2,TUBA1A,TUBA1C,TUBB3,TUBB4A	6
Cancer,Neurological Disease,Organismal Injury and Abnormalities	1p/19q co-deletion anaplastic glioma	4,92E-03			IDH1,IDH2,TUBA1A,TUBA1C,TUBB3,TUBB4A	6
Cancer,Endocrine System Disorders,Organismal Injury and Abnormalities,Reproductive System Diseas	Stage III-IV advanced ovarian carcinoma	4,92E-03			CD274,PARP2,TUBA1A,TUBA1C,TUBB3,TUBB4A	6
Cancer,Organismal Injury and Abnormalities,Reproductive System Disease	TNM stage T1-4 non-metastatic breast cancer	4,92E-03			PARP2,PARP3,TUBA1A,TUBA1C,TUBB3,TUBB4A	6
Cancer,Dermatological Diseases and Conditions,Hereditary Disorder,Metabolic Disease,Organismal In	Xeroderma pigmentosum	4,92E-03			ERCC1,ERCC2,ERCC5,ERCC6,GTPBP2,SMO	6
Cancer,Organismal Injury and Abnormalities,Tumor Morphology	Progressive PIK3CA mutation positive solid tumor	4,92E-03			AKT1,FKBP1A,TUBA1A,TUBA1C,TUBB3,TUBB4A	6
Cancer,Organismal Injury and Abnormalities	PTEN mutation positive solid tumor	4,92E-03			AKT1,FKBP1A,TUBA1A,TUBA1C,TUBB3,TUBB4A	6
Cancer,Neurological Disease,Organismal Injury and Abnormalities	1p/19q co-deletion positive oligodendroglioma	4,92E-03			IDH1,IDH2,TUBA1A,TUBA1C,TUBB3,TUBB4A	6
Cancer,Organismal Injury and Abnormalities	Low grade serous peritoneal adenocarcinoma	4,92E-03			MAP2K2,MAPK1,TUBA1A,TUBA1C,TUBB3,TUBB4A	6
Cancer,Organismal Injury and Abnormalities	Stage IV endometrioid cancer	4,92E-03			PARP2,PARP3,TUBA1A,TUBA1C,TUBB3,TUBB4A	6
Cancer,Endocrine System Disorders,Organismal Injury and Abnormalities,Reproductive System Diseas	Stage III-IV ovarian endometrioid carcinoma	4,92E-03			PARP2,PARP3,TUBA1A,TUBA1C,TUBB3,TUBB4A	6
Cancer,Endocrine System Disorders,Organismal Injury and Abnormalities,Reproductive System Diseas	Ovarian serous carcinosarcoma	4,92E-03			CD274,PARP2,TUBA1A,TUBA1C,TUBB3,TUBB4A	6
Cancer,Endocrine System Disorders,Organismal Injury and Abnormalities,Reproductive System Diseas	High grade advanced serous ovarian adenocarcin	4,92E-03			CD274,PARP2,TUBA1A,TUBA1C,TUBB3,TUBB4A	6
Cancer,Organismal Injury and Abnormalities,Respiratory Disease	Recurrent EGFR sensitizing mutation positive non	4,92E-03			CD274,TUBA1A,TUBA1C,TUBB3,TUBB4A,VEGFA	6
Cancer,Hereditary Disorder,Organismal Injury and Abnormalities	Hereditary cancer	5,03E-03			AKT1,AXIN2,AXL,BCL2L1,BRCA2,CD274,CHIC2,DDX41,EHBP1,EPHB2,ERCC2,ERCC6,FBXO11,FLCN,HNRN	40
Cancer,Endocrine System Disorders,Organismal Injury and Abnormalities	Secreting adrenocortical adenoma	5,11E-03			ALDH5A1,ASPN,BGN,COL6A1,COL6A2,DAD1,DDOST,EFEMP2,FLNC,MGAT1,OSTC,PRELP,RHOC,RPN2,S1	16
Cell Death and Survival	Apoptosis of breast cell lines	5,11E-03		0,834	AKT1,BAD,CDKN1B,DYNLL1,FOXO3,HRAS,IRF1,ITGA5,LIMS1,MAP3K5,MAPK1,MYC,TGFBR1,TIMP1,TNF:	16
Cancer,Organismal Injury and Abnormalities,Reproductive System Disease	Luminal breast carcinoma	5,22E-03			AKT1,BRCA2,EIF2S2,EIF6,PARP2,PARP3,SF3B1,TUBA1A,TUBA1C,TUBB3,TUBB4A	11
Metabolic Disease,Organismal Injury and Abnormalities	Oxidative phosphorylation deficiency	5,22E-03			AIFM1,COX15,GATB,GFM1,GFM2,MRPL3,MTFMT,PNPT1,SLC25A26,TARS2,TRIT1	11
Cancer,Organismal Injury and Abnormalities,Reproductive System Disease	Hormone receptor-positive HER2-negative breast	5,22E-03			AKT1,BRCA2,CHD1,FKBP1A,PARP2,PARP3,TUBA1A,TUBA1C,TUBB3,TUBB4A,VEGFA	11
Cancer,Organismal Injury and Abnormalities,Reproductive System Disease	Metastatic hormone receptor positive breast can	5,22E-03			AKT1,CD274,FKBP1A,HDAC10,PARP2,PARP3,TUBA1A,TUBA1C,TUBB3,TUBB4A,VEGFA	11
Cancer,Organismal Injury and Abnormalities,Reproductive System Disease	Recurrent hormone receptor positive HER2 negat	5,22E-03			AKT1,CHD1,FKBP1A,PARP2,PARP3,TUBA1A,TUBA1C,TUBB,TUBB3,TUBB4A,VEGFA	11
Dermatological Diseases and Conditions,Organismal Injury and Abnormalities	Psoriatic lesion	5,23E-03			BCL2L1,KRT18,STIM1,TNIP1	4
Cell Cycle	S phase of hematopoietic cell lines	5,23E-03			CCND3,MAP3K8,PNPT1,RBM5	4
Cancer,Organismal Injury and Abnormalities	Grade 2-3 endometrioid peritoneal cancer	5,23E-03			CD274,PARP2,PARP3,VEGFA	4
Cancer,Organismal Injury and Abnormalities,Reproductive System Disease	Grade 2-3 endometrioid fallopian tube cancer	5,23E-03			CD274,PARP2,PARP3,VEGFA	4
Cancer,Cell Death and Survival,Organismal Injury and Abnormalities,Tumor Morphology	Apoptosis of pancreatic cancer cells	5,23E-03			CASP1,EIF3F,IRF1,TNFSF10	4
Cardiovascular System Development and Function	Proliferation of vessel	5,23E-03			AKT1,ANGPT1,FOXO1,PF4	4
Dermatological Diseases and Conditions,Developmental Disorder,Hereditary Disorder,Metabolic Dise:	Cockayne syndrome	5,23E-03			ERCC1,ERCC2,ERCC5,ERCC6	4
Lipid Metabolism,Molecular Transport,Small Molecule Biochemistry,Vitamin and Mineral Metabolism	Quantity of 11-cis-retinal	5,23E-03		-1	CR1L,MAPK1,SLC27A4,VEGFA	4
Cell Cycle	Arrest in interphase of kidney cell lines	5,27E-03			CCND3,CDKN1B,MYC,OGT,PPP2CA,PPP2CB,RASSF1,RBBP6	8
Infectious Diseases	Infection of fibroblast cell lines	5,27E-03		-1,132	APOBEC3B,DAG1,HRAS,IFNAR1,IKBKE,MXRA8,RPS6KB2,TBK1	8
Cell Cycle	G1 phase of connective tissue cells	5,49E-03		-1,387	Cdc42,CDKN1B,CRKL,FOXC2,HRAS,MAP3K5,MAPK1,MYC,RAC1	9
Cancer,Organismal Injury and Abnormalities,Reproductive System Disease	Locally advanced mammary tumor	5,49E-03			AKT1,CD274,CHD1,FKBP1A,PARP2,TUBA1A,TUBA1C,TUBB3,TUBB4A	9
Cell Death and Survival	Apoptosis of tumor cell lines	5,52E-03		1,511	AGO1,AIFM1,AKT1,AKT1S1,ALKBH3,ANXA5,ARHGDIA,ATAD2,ATF5,ATN1,ATP1B3,BAD,BAG3,BCL2L1,B(	201
Cancer,Organismal Injury and Abnormalities,Reproductive System Disease	Invasive breast carcinoma	5,53E-03			AKT1,ATAD2,BCCIP,BCL2L1,BGN,BRCA2,CCND3,CD274,CHD1,CXCL10,DNM1L,ELP3,FBLN5,FKBP1A,FOX	45
Cancer,Hematological Disease,Organismal Injury and Abnormalities	Primary myelofibrosis	5,64E-03			AKT1,ANGPT1,ARHGAP18,BCL2L1,BMPR2,BRWD1,CD9,FOXO1,IDH1,IDH2,ITGA5,ITGB3,JARID2,MYC,PF	19
Dermatological Diseases and Conditions,Inflammatory Disease,Inflammatory Response,Organismal In	Juvenile dermatomyositis	5,77E-03			ASPN,BGN,CELF2,COL6A1,COL6A2,CYB561,LOXL1,LTBP3,PLOD2,RASSF1,SDC2,SDCBP	12
Cancer,Organismal Injury and Abnormalities	Stage I epithelial cancer	5,80E-03			ACTR10,AGO1,AGO2,ATIC,CHD1,EEF1A1,FKBP1A,KLK10,RHOC,RPL7,RPS11,RPS27A,SSR2,TUBA1A,TUB/	17
Cell Death and Survival	Cell death of fibroblasts	6,00E-03		0,889	AIFM1,AKT1,BAD,BCL2L1,CABLES2,CAPNS1,CASP1,CAT,Cdc42,CDKN1B,CHUK,CLIC4,CRKL,CYP1B1,EMD	48
Cell Cycle	G2/M phase transition	6,01E-03		-0,152	ARPP19,ATF5,Calm1 (includes others),CCP110,CENPJ,CEP135,CEP152,CEP290,CEP43,CNTRL,DYNLL1,FI	32
Cell Cycle	Arrest in interphase	6,02E-03			ACIN1,ACVRL1,ADRA1A,AKT1,ATF5,AXL,BAD,BAG3,BCL2L1,CAMK2N1,CAT,CAV1,CCND3,CDKN1B,Cdkn	75
Cancer,Organismal Injury and Abnormalities	High grade BRCA2 mutation positive peritoneal ca	6,12E-03			CD274,PARP2,PARP3,TUBA1A,TUBA1C,TUBB3,TUBB4A	7
Cancer,Endocrine System Disorders,Organismal Injury and Abnormalities,Reproductive System Diseas	Relapsed BRCA1 mutation positive serous ovarian	6,12E-03			CD274,PARP2,PARP3,TUBA1A,TUBA1C,TUBB3,TUBB4A	7
Cancer,Organismal Injury and Abnormalities	BRCA somatic mutation positive solid tumor	6,12E-03			CD274,PARP2,PARP3,TUBA1A,TUBA1C,TUBB3,TUBB4A	7
Cancer,Organismal Injury and Abnormalities	High grade BRCA1 mutation positive peritoneal ca	6,12E-03			CD274,PARP2,PARP3,TUBA1A,TUBA1C,TUBB3,TUBB4A	7
Cancer,Endocrine System Disorders,Organismal Injury and Abnormalities,Reproductive System Diseas	BRCA2 mutation positive serous ovarian adenoca	6,12E-03			CD274,PARP2,PARP3,TUBA1A,TUBA1C,TUBB3,TUBB4A	7
Cancer,Organismal Injury and Abnormalities,Reproductive System Disease	High grade BRCA2 mutation positive fallopian tub	6,12E-03			CD274,PARP2,PARP3,TUBA1A,TUBA1C,TUBB3,TUBB4A	7
Cancer,Organismal Injury and Abnormalities,Reproductive System Disease	High grade BRCA1 mutation positive fallopian tub	6,12E-03			CD274,PARP2,PARP3,TUBA1A,TUBA1C,TUBB3,TUBB4A	7
Cancer,Organismal Injury and Abnormalities	Invasive squamous-cell carcinoma	6,12E-03			CD274,SMAD4,TUBA1A,TUBA1C,TUBB3,TUBB4A,VEGFA	7
Cancer,Endocrine System Disorders,Hereditary Disorder,Organismal Injury and Abnormalities,Reprod	BRCA germline mutation positive serous ovarian	6,12E-03			CD274,PARP2,PARP3,TUBA1A,TUBA1C,TUBB3,TUBB4A	7
Cancer,Organismal Injury and Abnormalities,Respiratory Disease	Stage IIIB-IV recurrent nonsquamous non-small c	6,12E-03			ATIC,CD274,TUBA1A,TUBA1C,TUBB3,TUBB4A,VEGFA	7
Organismal Injury and Abnormalities	Dyskeratosis congenita or aplastic anemia	6,12E-03			BRCA2,CAT,DDX41,FKBP1A,GPX1,NHP2,PPP3R1,RPL18,RPL26,RPL35,RPL35A,RPS10,RPS15A,RPS19,RPS	20
Cancer,Organismal Injury and Abnormalities	Transitional-cell carcinoma	6,19E-03			AKT1,ARHGAP35,ASXL2,ATAD2,ATIC,AXIN2,AXL,BRCA2,CD274,CDKN1B,CLIP3,CSPG4,EIF4A2,ERCC2,FBI	53
Neurological Disease,Organismal Injury and Abnormalities	Leukoencephalopathy	6,30E-03			ABCD2,AIFM1,CAV1,COX15,DEGS1,DLD,FLOT2,GOLPH3,HIKESHI,IARS2,LRPPRC,MTFMT,NDUFS1,PHYH	22
Cardiovascular Disease,Organismal Injury and Abnormalities,Skeletal and Muscular Disorders	Dilated or arrhythmogenic cardiomyopathy	6,36E-03			ABCC9,ACE,ACE2,ADRA1A,AIFM1,ATP2A3,BAG3,BCL2L1,BGN,CAPNS1,CAV1,CEP85L,CIITA,DAG1,DNM:	48
Cancer,Organismal Injury and Abnormalities,Respiratory Disease	Stage I non-small cell lung carcinoma	6,40E-03			ACTR10,AGO1,AGO2,ATIC,EEF1A1,RHOC,RPL7,RPS11,RPS27A,SSR2,TUBA1A,TUBA1C,TUBB3,TUBB4A	14

Categories	Diseases or Functions Annotation	p-value	Predicted Activation State	Activation z-score	Molecules	# Molecules
Cancer,Neurological Disease,Organismal Injury and Abnormalities	Development of astrocytoma	6,40E-03			AKT1,ALDH5A1,ANGPT1,CSPG4,HRAS,IDH1,IDH2,MAP2K2,OAZ1,TUBA1A,TUBA1C,TUBB3,TUBB4A,VEG	14
Cancer,Endocrine System Disorders,Organismal Injury and Abnormalities,Reproductive System Diseas	Clear-cell ovarian carcinoma	6,53E-03			BRCA2,CD274,CLDN3,NOTCH3,PARP2,PARP3,PF4,PPP2R1A,SPINT2,STAT1,TUBA1A,TUBA1C,TUBB3,TU	15
Cancer,Organismal Injury and Abnormalities,Respiratory Disease	Stage I-II non-small cell lung cancer	6,53E-03			ACTR10,AGO1,AGO2,ATIC,CD274,EEF1A1,RHOC,RPL7,RPS11,RPS27A,SSR2,TUBA1A,TUBA1C,TUBB3,TU	15
Metabolic Disease,Organismal Injury and Abnormalities	Combined oxidative phosphorylation deficiency	6,55E-03			AIFM1,GATB,GFM1,GFM2,MRPL3,MTFMT,PNPT1,SLC25A26,TARS2,TRIT1	10
Connective Tissue Development and Function,Skeletal and Muscular System Development and Functi	Attachment of rib	6,55E-03			BMPR2,Cdkn1c,CHUK,DEAF1,GNAI2,GNAI3,LMO4,SF3B1,SLC29A1,WFIKKN2	10
Organ Morphology,Skeletal and Muscular System Development and Function	Morphology of skeletal muscle	6,59E-03			ADAM12,AKT1,CAV1,Cdkn1c,CFL2,CLIP3,COL6A1,DAG1,EIF2AK4,EMD,ERCC1,FLNC,FOXO1,HACD1,HAD	35
Cancer,Organismal Injury and Abnormalities,Renal and Urological Disease	Urothelial cancer	6,62E-03			AKT1,ARHGAP35,ASXL2,ATAD2,AXIN2,AXL,BRCA2,CCND3,CD274,CDKN1B,CIITA,CLIP3,CSPG4,DAD1,EIF	55
Cancer,Organismal Injury and Abnormalities	Stage I cancer	6,63E-03			ACTR10,AGO1,AGO2,ATIC,CHD1,CYP1B1,EEF1A1,FKBP1A,IFNAR1,KLK10,MAP2K2,MDM4,PSMB1,PSME	23
Cardiovascular Disease,Organismal Injury and Abnormalities,Respiratory Disease	Primary pulmonary hypertension	6,65E-03			ACVRL1,BCL2L1,BMPR2,CAV1,EIF2AK4,FOXO1,PFKFB3,SMAD4	8
Cancer,Organismal Injury and Abnormalities,Reproductive System Disease	Undifferentiated fallopian tube carcinoma	6,65E-03			CD274,PARP2,PARP3,TUBA1A,TUBA1C,TUBB3,TUBB4A,VEGFA	8
Cell Cycle,Embryonic Development	Arrest in G2 phase of embryonic cell lines	6,65E-03			CDKN1B,CKS1B,OGT,PPP2CA,PPP2CB,RASSF1,RBBP6,REV1	8
Cancer,Endocrine System Disorders,Organismal Injury and Abnormalities,Reproductive System Diseas	Stage IV HER2 negative hormone receptor negati	6,65E-03			CD274,CHD1,TUBA1A,TUBA1C,TUBB,TUBB3,TUBB4A,VEGFA	8
Cancer,Endocrine System Disorders,Organismal Injury and Abnormalities,Reproductive System Diseas	Recurrent ovarian endometrioid carcinoma	6,65E-03			CD274,PARP2,PARP3,TUBA1A,TUBA1C,TUBB3,TUBB4A,VEGFA	8
Cancer,Endocrine System Disorders,Organismal Injury and Abnormalities,Reproductive System Diseas	Recurrent undifferentiated ovarian carcinoma	6,65E-03			CD274,PARP2,PARP3,TUBA1A,TUBA1C,TUBB3,TUBB4A,VEGFA	8
Cancer,Organismal Injury and Abnormalities,Respiratory Disease	Mixed cell non-small cell lung carcinoma	6,74E-03			AKT1,ATIC,CD274,CSPG4,RPS15,TUBA1A,TUBA1C,TUBB3,TUBB4A	9
Cancer,Organismal Injury and Abnormalities,Respiratory Disease	Stage IV stage IV lung squamous cell carcinoma	6,74E-03			ATIC,CD274,PARP2,PARP3,TUBA1A,TUBA1C,TUBB3,TUBB4A,VEGFA	9
Cancer,Organismal Injury and Abnormalities,Reproductive System Disease	HER2-negative breast carcinoma	6,76E-03			AKT1,BRCA2,CD274,CHD1,FKBP1A,PARP2,PARP3,TUBA1A,TUBA1C,TUBB3,TUBB4A,VEGFA	12
Cancer,Organismal Injury and Abnormalities,Reproductive System Disease	Locally advanced breast cancer	6,76E-03			AKT1,CD274,CHD1,FKBP1A,HDAC10,PARP2,PARP3,TUBA1A,TUBA1C,TUBB,TUBB3,TUBB4A	12
Cancer,Endocrine System Disorders,Organismal Injury and Abnormalities,Reproductive System Diseas	BRCA1 mutation negative BRCA2 mutation negati	6,76E-03			PARP2,PARP3,TUBA1A,TUBA1C,TUBB3,TUBB4A	6
Cancer,Endocrine System Disorders,Organismal Injury and Abnormalities,Reproductive System Diseas	Stage 3C ovarian carcinoma	6,76E-03			KLK10,TUBA1A,TUBA1C,TUBB3,TUBB4A,VEGFA	6
Neurological Disease,Organismal Injury and Abnormalities	Recurrent encephalopathy	6,76E-03			MYC,RHEB,TUBA1A,TUBA1C,TUBB3,TUBB4A	6
Cancer,Organismal Injury and Abnormalities,Respiratory Disease	Unresectable EGFR exon 19 deletion negative nor	6,76E-03			PARP2,TUBA1A,TUBA1C,TUBB3,TUBB4A,VEGFA	6
Cancer,Organismal Injury and Abnormalities,Respiratory Disease	Advanced EGFR mutation negative ALK rearrange	6,76E-03			CD274,TUBA1A,TUBA1C,TUBB3,TUBB4A,VEGFA	6
Cell-mediated Immune Response,Cellular Development,Cellular Function and Maintenance,Cellular Gı	Proliferation of double-negative T lymphocyte	6,76E-03		-0,447	CDKN1B,HNRNPL,MAP3K14,RASGRP3,RICTOR,TNFAIP3	6
Cell Death and Survival	Activation-induced cell death of T-cell hybrid cells	6,77E-03		-1,432	AKT1,DNAJA3,RELA,RORC,TNFAIP3	5
Cell Cycle	G1/S phase transition of connective tissue cells	6,77E-03		-1	Cdc42,CDKN1B,FOXC2,HRAS,RAC1	5
Skeletal and Muscular Disorders	Vacuolar myopathy	6,77E-03			CASQ1,NBR1,PSMA2,PSMB5,SQSTM1	5
Behavior,Nervous System Development and Function	Context memory	6,77E-03		1,067	ACTG1,GADD45G,MAPK8IP1,OGT,QPCT	5
Cell Death and Survival	Apoptosis of breast cancer cell lines	6,88E-03		1,151	AGO1,AKT1,ARHGDIA,BAD,BAG3,BCL2L1,BCL2L12,BMX,CASP1,CAT,CAV1,CD274,CKS1B,DNAJA3,EHMT	53
Cell Death and Survival	Apoptosis of melanoma cell lines	6,92E-03		1,287	AKT1S1,BAG3,BCL2L1,CDKN1B,DYNLL1,EIF3F,FBXO11,FOXO3,GADD45G,GBA2,HSPB8,MAP3K5,MAPK1	24
Cancer,Organismal Injury and Abnormalities	Benign solid tumor	7,03E-03		-1,325	ADAM12,AIG1,AKAP8L,AKT1,ALDH5A1,ANAPC10,ANGPT1,ANTXR1,ANXA5,ASPN,ATP6V1F,AXIN2,AXL,E	151
Cellular Development,Cellular Growth and Proliferation,Hair and Skin Development and Function	Proliferation of epithelial cell lines	7,19E-03		-0,859	ABHD8,ADRA1A,AKAP8L,AKT1,BRCA2,CAT,CDKN1B,CSTF2,EIF3E,FBLN5,FOXC2,FOXO1,FOXO3,GNAI2,H	43
Infectious Diseases	Infection by HIV-1	7,31E-03	Decreased	-3,745	ACTR3,AFG3L2,AKT1,ALKBH3,ANKRD6,ASXL2,ATG16L2,ATP6V0A1,BCLAF1,BCR,CAPN6,CCT2,CD164,CL	95
Cancer,Organismal Injury and Abnormalities,Reproductive System Disease	Stage IV advanced breast cancer	7,33E-03			CD274,CHD1,FKBP1A,PARP2,PARP3,TUBA1A,TUBA1C,TUBB,TUBB3,TUBB4A,VEGFA	11
Cancer,Endocrine System Disorders,Organismal Injury and Abnormalities,Reproductive System Diseas	Advanced HER2 negative hormone receptor posit	7,33E-03			AKT1,CHD1,FKBP1A,PARP2,PARP3,TUBA1A,TUBA1C,TUBB,TUBB3,TUBB4A,VEGFA	11
Cancer,Hematological Disease,Immunological Disease	Lymphoid hyperplasia	7,33E-03		-0,915	AKT1,AXL,FOXO3,ICOSLG/LOC102723996,IFNGR1,IL15RA,MALT1,MAP3K14,TCIM,TNFRSF21,TRAF3IP2	11
Cancer,Endocrine System Disorders,Organismal Injury and Abnormalities	Aldosterone producing adrenocortical adenoma	7,42E-03			ALDH5A1,ASPN,BGN,COL6A1,COL6A2,DAD1,DDOST,EFEMP2,FLNC,MGAT1,OSTC,PRELP,RHOC,RPN2,ST	15
Cell Death and Survival,Skeletal and Muscular Disorders	Cell death of muscle cell lines	7,42E-03		0,393	ANGPT1,BAD,BCL2L1,CAV1,EEF1A1,HSPB6,IRF1,LAMP2,NOTCH3,RAC1,SH3BP5,SIVA1,TNFRSF10A,TNF	15
Hereditary Disorder,Organismal Injury and Abnormalities,Skeletal and Muscular Disorders	Autosomal dominant myopathy	7,45E-03			ABCC9,ASPN,BAG3,BGN,CASQ1,CCND3,CELF2,CNBP,COL6A1,COL6A2,CYB561,DDX5,DNM1L,EMD,FLN	39
Cancer,Organismal Injury and Abnormalities	Neoplasia of fibroblast cell lines	7,62E-03		-0,654	AFAP1L1,AKT1,BATF2,BRD7,CAV1,Cdc42,DYNLL1,EEF1A1,GNAI2,HNRNPA2B1,HRAS,IRF1,MAP3K8,MA	22
Organ Morphology,Organismal Injury and Abnormalities,Skeletal and Muscular Disorders,Skeletal anc	Abnormal morphology of skeletal muscle	7,62E-03			ADAM12,CAV1,Cdkn1c,CFL2,CLIP3,COL6A1,EMD,FLNC,HADHA,Hnrnpa1,HSPB8,ILF3,LMO7,MEOX2,Ptp	22
Cancer,Organismal Injury and Abnormalities	Carcinoma of unknown primary	7,78E-03			AKT1,ASXL2,AXIN2,CD274,CHD1,COP1,FLCN,IDH1,MAPK1,MDM4,NOTCH3,SETD2,TET2,TNFAIP3,TUBA	19
Metabolic Disease,Organismal Injury and Abnormalities,Renal and Urological Disease	Organic aciduria	7,87E-03			DBT,DLD,ETFDH,IDH1,IDH2,IVD,MCCC1,MCCC2,PCCB,PPM1K,SUCLA2,SUGCT	12
Cancer,Organismal Injury and Abnormalities,Respiratory Disease	Stage I metastatic non-small cell lung carcinoma	7,89E-03			ACTR10,EEF1A1,RHOC,RPL7,RPS11,RPS27A,SSR2	7
Cancer,Hematological Disease,Immunological Disease,Organismal Injury and Abnormalities	Stage II multiple myeloma	7,89E-03			IFNAR1,PSMB1,PSMB5,TUBA1A,TUBA1C,TUBB3,TUBB4A	7
Cancer,Hematological Disease,Immunological Disease,Organismal Injury and Abnormalities	Stage III multiple myeloma	7,89E-03			IFNAR1,PSMB1,PSMB5,TUBA1A,TUBA1C,TUBB3,TUBB4A	7
Cancer,Hereditary Disorder,Organismal Injury and Abnormalities	Primary BRCA2 germline mutation positive perito	7,89E-03			CD274,PARP2,PARP3,TUBA1A,TUBA1C,TUBB3,TUBB4A	7
Cancer,Organismal Injury and Abnormalities,Respiratory Disease,Tumor Morphology	Stage 4 progressive EGFR sensitizing mutation po	7,89E-03			ATIC,CD274,TUBA1A,TUBA1C,TUBB3,TUBB4A,VEGFA	7
Cell Morphology,Cellular Function and Maintenance	Autophagy of colorectal cancer cell lines	7,89E-03		0,218	BCL2L1,CHUK,FOXO1,GABARAPL1,GNAI3,MYC,XBP1	7
Cancer,Endocrine System Disorders,Hereditary Disorder,Organismal Injury and Abnormalities,Reprod	BRCA1 germline mutation positive ovarian adeno	7,89E-03			CD274,PARP2,PARP3,TUBA1A,TUBA1C,TUBB3,TUBB4A	7
Cancer,Hereditary Disorder,Organismal Injury and Abnormalities,Reproductive System Disease	BRCA2 germline mutation positive fallopian tube	7,89E-03			CD274,PARP2,PARP3,TUBA1A,TUBA1C,TUBB3,TUBB4A	7
Cell Death and Survival	Cell survival of lung cancer cell lines	7,89E-03		0,875	BCL2L1,FBXO5,HTRA3,IKBKE,PSMB4,TUBB3,UBE2L3	7
Cancer,Organismal Injury and Abnormalities,Respiratory Disease	EGFR activating mutation positive EGFR T790M m	7,89E-03			ATIC,CD274,TUBA1A,TUBA1C,TUBB3,TUBB4A,VEGFA	7
Cancer,Organismal Injury and Abnormalities	Residual cancer	7,89E-03			CD274,PARP2,TUBA1A,TUBA1C,TUBB3,TUBB4A,VEGFA	7
Cancer,Organismal Injury and Abnormalities,Reproductive System Disease	Primary HER2 negative hormone receptor negativ	7,89E-03			CHD1,PARP2,PARP3,TUBA1A,TUBA1C,TUBB3,TUBB4A	7
Cancer,Endocrine System Disorders,Organismal Injury and Abnormalities,Reproductive System Diseas	Locally advanced HER2-negative breast carcinom	7,89E-03			AKT1,CD274,FKBP1A,TUBA1A,TUBA1C,TUBB3,TUBB4A	7
Cancer,Neurological Disease,Organismal Injury and Abnormalities	Diffuse glioma	7,89E-03			ALDH5A1,BRCA2,H3-3A/H3-3B,IDH1,IDH2,MAP2K2,VEGFA	7
Cancer,Organismal Injury and Abnormalities	BRCA1 mutation positive endometrioid cancer	7,89E-03			CD274,PARP2,PARP3,TUBA1A,TUBA1C,TUBB3,TUBB4A	7
Cancer,Hereditary Disorder,Organismal Injury and Abnormalities,Reproductive System Disease	Invasive familial breast cancer	7,89E-03			BRCA2,PARP2,PARP3,TUBA1A,TUBA1C,TUBB3,TUBB4A	7
Cancer,Endocrine System Disorders,Hereditary Disorder,Organismal Injury and Abnormalities,Reprod	BRCA2 germline mutation positive ovarian adeno	7,89E-03			CD274,PARP2,PARP3,TUBA1A,TUBA1C,TUBB3,TUBB4A	7
Cancer,Hereditary Disorder,Organismal Injury and Abnormalities	Primary BRCA1 germline mutation positive perito	7,89E-03			CD274,PARP2,PARP3,TUBA1A,TUBA1C,TUBB3,TUBB4A	7
Cancer,Organismal Injury and Abnormalities	BRCA2 mutation positive endometrioid cancer	7,89E-03			CD274,PARP2,PARP3,TUBA1A,TUBA1C,TUBB3,TUBB4A	7
Cancer,Hereditary Disorder,Organismal Injury and Abnormalities,Reproductive System Disease	BRCA1 germline mutation positive fallopian tube	7,89E-03			CD274,PARP2,PARP3,TUBA1A,TUBA1C,TUBB3,TUBB4A	7
Cardiovascular Disease,Cardiovascular System Development and Function,Organ Morphology,Organis	Dilated cardiomyopathy	8,08E-03			ABCC9,ACE,ACE2,ADRA1A,AIFM1,ATP2A3,BAG3,BCL2L1,BGN,CAPNS1,CAV1,CEP85L,CIITA,DAG1,DNM1	47
Cancer,Hereditary Disorder,Organismal Injury and Abnormalities,Renal and Urological Disease	Hereditary urinary system neoplasm	8,19E-03			BCL2L1,BRCA2,FLCN,NFE2L2,RHEB,SETD2,SF3B1,TRAP1,VEGFA	9
Cell Death and Survival	Cell death of astrocytes	8,19E-03		-1,179	BCL2L1,BCL2L12,DNM1L,MAPK1,NFE2L2,PFKFB3,RPS6KB2,SH3KBP1,TNFSF10	9

Categories	Diseases or Functions Annotation	p-value	Predicted Activation State	Activation z-score	Molecules	# Molecules
Cancer,Endocrine System Disorders,Organismal Injury and Abnormalities,Reproductive System Diseas	Metastatic hormone receptor positive HER2 nega	8,19E-03			AKT1,FKBP1A,PARP2,PARP3,TUBA1A,TUBA1C,TUBB3,TUBB4A,VEGFA	9
Cancer,Organismal Injury and Abnormalities	Platinum resistant primary peritoneal cancer	8,28E-03			CD274,PARP2,PARP3,TUBA1A,TUBA1C,TUBB3,TUBB4A,VEGFA	8
Cancer,Organismal Injury and Abnormalities,Reproductive System Disease	High grade serous fallopian tube carcinoma	8,28E-03			CD274,PARP2,PARP3,TUBA1A,TUBA1C,TUBB3,TUBB4A,VEGFA	8
Cancer,Endocrine System Disorders,Organismal Injury and Abnormalities,Reproductive System Diseas	Stage III-IV advanced ovarian cancer	8,28E-03			CD274,PARP2,PARP3,TUBA1A,TUBA1C,TUBB3,TUBB4A,VEGFA	8
Cancer,Organismal Injury and Abnormalities,Reproductive System Disease	Metastatic HER2-negative breast carcinoma	8,28E-03			AKT1,CD274,FKBP1A,TUBA1A,TUBA1C,TUBB3,TUBB4A,VEGFA	8
Cancer,Organismal Injury and Abnormalities,Reproductive System Disease	Clear cell malignant fallopian tube tumor	8,28E-03			CD274,PARP2,PARP3,TUBA1A,TUBA1C,TUBB3,TUBB4A,VEGFA	8
Cancer,Organismal Injury and Abnormalities	Clear cell malignant peritoneal tumor	8,28E-03			CD274,PARP2,PARP3,TUBA1A,TUBA1C,TUBB3,TUBB4A,VEGFA	8
Cell Cycle,Connective Tissue Development and Function	G1 phase of fibroblasts	8,28E-03		-1,067	Cdc42,CDKN1B,CRKL,HRAS,MAP3K5,MAPK1,MYC,RAC1	8
Cancer,Organismal Injury and Abnormalities,Reproductive System Disease	HER2 negative hormone receptor negative invasi	8,28E-03			CD274,CHD1,PARP2,PARP3,TUBA1A,TUBA1C,TUBB3,TUBB4A	8
Cell Morphology	Shape change of cervical cancer cell lines	8,28E-03	Decreased	-2,6	AKT1,GIT2,MAP3K5,MAPRE3,MYADM,PARVA,RAC1,UCHL1	8
Cancer,Organismal Injury and Abnormalities,Reproductive System Disease	Refractory fallopian tube cancer	8,28E-03			CD274,PARP2,PARP3,TUBA1A,TUBA1C,TUBB3,TUBB4A,VEGFA	8
Cellular Development,Cellular Growth and Proliferation,Connective Tissue Development and Function	Proliferation of fibroblast cell lines	8,31E-03		-1,282	ACAT1,ADRA1A,AKT1,AXL,BCL2L1,BRPF1,CAT,CAV1,CD9,CDKN1B,CIRBP,CKS1B,CSNK2B,CSTF2,CXCL10,	71
Cancer,Organismal Injury and Abnormalities,Reproductive System Disease	Hormone receptor positive breast carcinoma	8,34E-03			AKT1,BRCA2,CD274,CHD1,EIF2S2,EIF6,FKBP1A,MAP3K1,PARP2,PARP3,SF3B1,TUBA1A,TUBA1C,TUBB3,	16
DNA Replication, Recombination, and Repair	Transcription-coupled nucleotide-excision repair	8,36E-03			CUL4A,DDB1,ERCC1,ERCC2,ERCC5,ERCC6,POLD3,POLR2D,POLR2G,POLR2I,RFC1,RPS27A,TCEA1,UVSSA	14
Cancer,Organismal Injury and Abnormalities,Reproductive System Disease	Fallopian tube cancer	8,36E-03			ANGPT1,ATIC,BRCA2,CD274,CHD1,MAP2K2,MAPK1,PARP2,PARP3,TUBA1A,TUBA1C,TUBB3,TUBB4A,VE	14
Cell Death and Survival	Cell death of lung cancer cell lines	8,40E-03		0,455	AIFM1,AKT1,ATAD2,ATP1B3,AXL,BCL2L1,BRCA2,CASP1,CAV1,CCAR2,CCP110,CD274,CDKN1B,CLPTM1L	48
Cell Cycle	G0/G1 phase transition	8,40E-03			BAD,BCL2L1,CAV1,CDKN1B,FOXO3,HRAS,MAP3K5,MEOX2,MYC,RAC1,RACK1,RXRA,SDC2,STAT1,THRB	15
Developmental Disorder,Endocrine System Disorders,Hereditary Disorder,Organismal Injury and Abnc	Klinefelter syndrome	8,40E-03			FLNA,GSTM2,GTPBP6,ITGB3,LAMB2,MAP1A,MVB12B,PIK3R3,PKHD1L1,SMC1A,TXLNG,UBA1,USP31,W	15
Cell Morphology,Organ Morphology,Skeletal and Muscular System Development and Function,Tissue	Size of muscle cells	8,50E-03			ACE,ADRA1A,AKT1,CAPNS1,Cdkn1c,FLNC,FOXO1,FOXO3,FTO,HRAS,LIMS1,MAP3K1,MEOX2,MYC,NPR1	25
Developmental Disorder,Embryonic Development,Organismal Development,Tissue Morphology	Lack of mesoderm	8,61E-03			BMPR2,DAG1,DHX9,DRAP1,PPP2CA,RAC1,SMAD4,SRF,TRIM28,UBIAD1,WLS	11
Cancer,Organismal Injury and Abnormalities	High grade recurrent primary peritoneal serous a	8,67E-03			CD274,PARP2,PARP3,VEGFA	4
Cancer,Organismal Injury and Abnormalities	High grade primary recurrent peritoneal cancer	8,67E-03			CD274,PARP2,PARP3,VEGFA	4
Cancer,Endocrine System Disorders,Hereditary Disorder,Organismal Injury and Abnormalities,Reprod	Familial epithelial ovarian cancer	8,67E-03			BRCA2,PARP2,PARP3,VEGFA	4
Cellular Assembly and Organization	Formation of membrane rafts	8,67E-03			CAV1,Cdc42,RAC1,TNFSF10	4
Cancer,Organismal Injury and Abnormalities,Tumor Morphology	High grade progressive peritoneal serous cancer	8,67E-03			CD274,PARP2,PARP3,VEGFA	4
Organ Morphology,Organismal Injury and Abnormalities,Skeletal and Muscular Disorders,Skeletal anc	Abnormal morphology of intercostal muscle	8,67E-03			COL6A1,FLNC,Hnrnpa1,ILF3	4
Cancer,Organismal Injury and Abnormalities,Reproductive System Disease,Tumor Morphology	Progressive endometrioid fallopian tube cancer	8,67E-03			CD274,PARP2,PARP3,VEGFA	4
Cancer,Organismal Injury and Abnormalities,Tumor Morphology	Progressive endometrioid peritoneal cancer	8,67E-03			CD274,PARP2,PARP3,VEGFA	4
Cell Death and Survival	Apoptosis of schwannoma cell lines	8,84E-03			MYC,TNFRSF10A,TNFSF10	3
Cell-To-Cell Signaling and Interaction	Adhesion of E. coli	8,84E-03			CD151,CD63,CD9	3
Carbohydrate Metabolism	Metabolism of fructose-2,6-diphosphate	8,84E-03			PFKFB1,PFKFB2,PFKFB3	3
Cell-To-Cell Signaling and Interaction,Embryonic Development	Adhesion of embryonic stem cells	8,84E-03			CD9,ITGA5,SRF	3
Cell-To-Cell Signaling and Interaction	Breakdown of intercellular junctions	8,84E-03			CRKL,ITGA5,RAC1	3
Connective Tissue Development and Function,Embryonic Development,Organ Morphology,Organism:	Length of humerus	8,84E-03			Cdc42,GPC6,SMO	3
Cardiovascular Disease,Cardiovascular System Development and Function,Organ Morphology,Organis	Abnormal morphology of myocardial cuff	8,84E-03			AKT1,CDKN1B,EMD	3
Carbohydrate Metabolism	Quantity of fructose-2,6-diphosphate	8,84E-03			PFKFB1,PFKFB2,PFKFB3	3
Infectious Diseases	Infection of fibrosarcoma cell lines	8,84E-03			AXL,STAT1,STAT2	3
Cardiovascular System Development and Function	Proliferation of blood vessel	8,84E-03			AKT1,FOXO1,PF4	3
Developmental Disorder,Hereditary Disorder,Metabolic Disease,Organismal Injury and Abnormalities,	Maple syrup urine disease	8,84E-03			DBT,DLD,PPM1K	3
Cell Morphology	Elongation of embryonic cell lines	8,84E-03			CAV1,Cdc42,RAC1	3
Carbohydrate Metabolism,Small Molecule Biochemistry	Catabolism of D-glucose	8,84E-03			BAD,MYC,PFKFB2	3
Cancer,Organismal Injury and Abnormalities,Tumor Morphology	Progression of adenoma	8,84E-03			FOXO1,FOXO3,TSC1	3
Cell-To-Cell Signaling and Interaction	Binding of Escherichia coli 25922	8,84E-03			CD151,CD63,CD9	3
Amino Acid Metabolism,Small Molecule Biochemistry	Catabolism of leucine	8,84E-03			IVD,MCCC1,MCCC2	3
Developmental Disorder,Neurological Disease,Ophthalmic Disease,Organismal Injury and Abnormaliti	Congenital fibrosis of extraocular muscles	8,84E-03			KIF21A,TUBA1A,TUBB3	3
Cardiovascular Disease,Organismal Injury and Abnormalities	Rupture of aorta	8,84E-03			BGN,EFEMP2,LTBP3	3
Cell Cycle	Delay in initiation of G0/G1 phase transition	8,84E-03			BAD,BCL2L1,STAT1	3
Cellular Assembly and Organization,Cellular Function and Maintenance	Regrowth of microtubules	8,84E-03			CLIP3,MAPRE1,TBCB	3
Developmental Disorder,Hereditary Disorder,Metabolic Disease,Neurological Disease,Organismal Inju	Intellectual disability and inborn errors of metabc	8,84E-03			DLD,ERCC6,PDHX	3
Carbohydrate Metabolism,Small Molecule Biochemistry	Metabolism of isocitric acid	8,84E-03			IDH1,IDH3B,IDH3G	3
Small Molecule Biochemistry	Concentration of fructose-2,6-diphosphate	8,84E-03			HRAS,PFKFB2,PFKFB3	3
Cellular Development	Epithelial-mesenchymal transition of liver cell line	8,84E-03			BTBD7,FTH1,SMAD4	3
Cell Death and Survival,Skeletal and Muscular Disorders	Necrosis of muscle	9,01E-03		1,454	ABCC9,ACE2,ADAM12,ADRA1A,AKT1,ANGPT1,ATP2B4,BAD,BAG3,BCL2L1,BRCA2,C1QTNF9B,CASP1,CA	57
Cancer,Organismal Injury and Abnormalities,Respiratory Disease	Recurrent metastatic non-squamous non-small ci	9,05E-03			ATIC,TUBA1A,TUBA1C,TUBB3,TUBB4A,VEGFA	6
Cell Cycle,Hair and Skin Development and Function	G2 phase of epithelial cell lines	9,05E-03			MYC,OGT,PPP2CA,PPP2CB,RASSF1,RBBP6	6
Cancer,Organismal Injury and Abnormalities,Respiratory Disease	Unresectable EGFR sensitizing mutation positive i	9,05E-03			CD274,TUBA1A,TUBA1C,TUBB3,TUBB4A,VEGFA	6
Cancer,Organismal Injury and Abnormalities	Squamous cell carcinoma of unknown primary	9,05E-03			CD274,TUBA1A,TUBA1C,TUBB3,TUBB4A,VEGFA	6
RNA Post-Transcriptional Modification	Processing of RNA	9,08E-03		-0,342	AGO2,AKAP8L,C1D,CCAR2,CDK13,CELF2,CLK1,CLK2,CLK4,CPSF1,CSTF2,DDX23,DDX41,DDX5,DHX9,EM(	66
Infectious Diseases	Infection by RNA virus	9,29E-03	Decreased	-4,622	ACE,ACE2,ACTR3,ADRA1A,AFG3L2,AHSG,AKT1,ALKBH3,ANKRD6,AP2A1,APOBEC3B,ARHGAP12,ASXL2,/	167
Cell Death and Survival	Cell viability of tumor cell lines	9,40E-03		-1,136	ACE,ACVRL1,ADRM1,AGO2,AIFM1,AKAP8L,AKT1,ALKBH3,AMFR,ANAPC11,ANTXR1,ATF5,AXL,BAD,BAG	157
Metabolic Disease,Organismal Injury and Abnormalities,Renal and Urological Disease	Aciduria	9,51E-03			ACADL,DBT,DLD,ETFDH,IDH1,IDH2,IVD,LDHD,MCCC1,MCCC2,PCCB,PPM1K,SUCLA2,SUGCT	14
Hereditary Disorder,Organismal Injury and Abnormalities	Turner syndrome	9,54E-03			GRIPAP1,GSTM2,GTPBP6,ITGB3,LAMB2,MAGEE1,MAP1A,MVB12B,PIK3R3,PKHD1L1,PRAF2,RTL8C,SH3	19
Cardiovascular Disease,Cardiovascular System Development and Function,Developmental Disorder,O	Interrupted aortic arch	9,66E-03			BMPR2,CRKL,FLNA,FOXC2,VEGFA	5
Cancer,Organismal Injury and Abnormalities	Stage I-II cancer	9,82E-03			ACTR10,AGO1,AGO2,ATIC,CD274,CHD1,CYP1B1,EEF1A1,FKBP1A,IFNAR1,IL2RG,KLK10,LAMC2,MAP2K2	29
Cancer,Organismal Injury and Abnormalities,Reproductive System Disease	Multifocal breast cancer	9,88E-03			CD274,CHD1,FKBP1A,PARP2,PARP3,TUBA1A,TUBA1C,TUBB3,TUBB4A	9
Cancer,Organismal Injury and Abnormalities,Reproductive System Disease	Metastatic castration resistant prostatic adenoca	9,88E-03			AKT1,AXL,CD274,PARP2,PARP3,TUBA1A,TUBA1C,TUBB3,TUBB4A	9

Categories	Diseases or Functions Annotation	p-value	Predicted Activation State	Activation z-score	Molecules	# Molecules
Cancer,Organismal Injury and Abnormalities	Cystadenocarcinoma	9,88E-03			DYNLRB1,HRAS,PPP2R1A,RPS19,TUBA1A,TUBA1C,TUBB3,TUBB4A,VEGFA	9
Cancer,Organismal Injury and Abnormalities,Reproductive System Disease	Refractory breast cancer	9,88E-03			CD274,CHD1,FKBP1A,PARP2,PARP3,TUBA1A,TUBA1C,TUBB3,TUBB4A	9
Cancer,Organismal Injury and Abnormalities	BRCA1 mutation positive carcinoma	9,88E-03			ATIC,CD274,PARP2,PARP3,TUBA1A,TUBA1C,TUBB3,TUBB4A,VEGFA	9
Cancer,Organismal Injury and Abnormalities,Reproductive System Disease	Metastatic cervical neoplasm	9,88E-03			ALDH5A1,AOC3,ATIC,CD274,TUBA1A,TUBA1C,TUBB3,TUBB4A,VEGFA	9
Cancer,Organismal Injury and Abnormalities,Respiratory Disease	Stage IV metastatic non-squamous non-small cell	9,88E-03			ATIC,CD274,PARP2,PARP3,TUBA1A,TUBA1C,TUBB3,TUBB4A,VEGFA	9
Developmental Disorder,Hereditary Disorder,Organismal Injury and Abnormalities,Skeletal and Musc	Duchenne muscular dystrophy	9,90E-03			ACE,ADRA1A,ASPN,BGN,CELF2,COL6A1,COL6A2,CYB561,HLA-A,LOXL1,LTBP3,PKP2,PLOD2,RASSF1,SDC	18
Cell Cycle	Senescence of cells	9,90E-03		1,1	AKT1,ARHGAP18,BCL2L1,BCL2L12,BRD7,BUB3,CAT,CAV1,CDKN1B,CIRBP,CKS1B,DPY30,ERCC1,Ewsr1,F	55
Cancer,Organismal Injury and Abnormalities,Reproductive System Disease	HER2 negative hormone receptor positive invasiv	1,00E-02			CHD1,PARP2,PARP3,TUBA1A,TUBA1C,TUBB3,TUBB4A	7
Connective Tissue Disorders,Organismal Injury and Abnormalities,Skeletal and Muscular Disorders	Osteosclerosis	1,00E-02		1,432	ERCC2,IDS,ITGB3,LTBP3,POLR3B,THRA,THRB	7
Cell Cycle	Quiescence of blood cells	1,00E-02	Increased	2,599	CDKN1B,CKS1B,Meis1,MYC,TNFAIP3,TSC1,XRCC6	7
Cancer,Organismal Injury and Abnormalities,Reproductive System Disease	Recurrent HER2 negative hormone receptor posit	1,00E-02			AKT1,FKBP1A,TUBA1A,TUBA1C,TUBB3,TUBB4A,VEGFA	7
Cell Cycle	Exit from interphase	1,00E-02			CAV1,CDKN1B,CSNK2B,FOXO3,MYC,RASSF1,RBM5	7
Metabolic Disease,Neurological Disease,Organismal Injury and Abnormalities,Skeletal and Muscular D	Mitochondrial encephalomyopathy	1,01E-02			AIFM1,COX15,DLD,GOLPH3,IARS2,LRPPRC,MRPL3,MTFMT,NDUFS1,SCO1,SURF1	11
Cancer,Organismal Injury and Abnormalities,Reproductive System Disease	HER2 negative hormone receptor positive invasiv	1,02E-02			CHD1,FKBP1A,PARP2,PARP3,TUBA1A,TUBA1C,TUBB3,TUBB4A	8
Cancer,Organismal Injury and Abnormalities,Reproductive System Disease	Hormone receptor positive metastatic breast car	1,02E-02			AKT1,CD274,FKBP1A,TUBA1A,TUBA1C,TUBB3,TUBB4A,VEGFA	8
Cancer,Organismal Injury and Abnormalities,Reproductive System Disease	Stage IV estrogen receptor negative invasive brea	1,02E-02			CD274,CHD1,TUBA1A,TUBA1C,TUBB,TUBB3,TUBB4A,VEGFA	8
Embryonic Development,Organismal Development,Tissue Morphology	Morphology of ectoderm	1,02E-02			CR1L,DAG1,IL11RA,MAPK1,PIKFYVE,PTGIS,RAB10,SMAD4	8
Cancer,Organismal Injury and Abnormalities,Reproductive System Disease	Metastatic breast adenocarcinoma	1,02E-02			AKT1,CD274,FKBP1A,TUBA1A,TUBA1C,TUBB3,TUBB4A,VEGFA	8
Cancer,Organismal Injury and Abnormalities,Reproductive System Disease	Primary invasive breast adenocarcinoma	1,02E-02			CHD1,PARP2,PARP3,RASSF1,TUBA1A,TUBA1C,TUBB3,TUBB4A	8
Gastrointestinal Disease,Hepatic System Disease,Organismal Injury and Abnormalities	Degeneration of liver	1,02E-02		-0,053	AXL,CHUK,ERCC1,GIMAP1-GIMAP5,Mt2,PHYH,RELA,TBK1	8
Cancer,Connective Tissue Disorders,Organismal Injury and Abnormalities,Skeletal and Muscular Disor	Chondroma in phalanx	1,02E-02			IDH1,IDH2	2
Endocrine System Disorders,Metabolic Disease,Organismal Injury and Abnormalities	Acquired childhood hypothyroidism	1,02E-02			THRA,THRB	2
Developmental Disorder,Hereditary Disorder,Neurological Disease,Organismal Injury and Abnormaliti	Cerebrooculofacioskeletal syndrome 1	1,02E-02			ERCC2,ERCC6	2
Cell Death and Survival	Apoptosis of cortical astrocytes	1,02E-02			BCL2L12,NFE2L2	2
Developmental Disorder,Hereditary Disorder,Neurological Disease,Organismal Injury and Abnormaliti	Chromosome 9q subtelomeric deletion syndrome	1,02E-02			ABCC9,EHMT1	2
Cell Cycle,Hair and Skin Development and Function	Arrest in G2/M phase transition of keratinocytes	1,02E-02			MAP3K5,MYC	2
Cancer,Connective Tissue Disorders,Organismal Injury and Abnormalities,Skeletal and Muscular Disor	Chondrosarcoma in femur	1,02E-02			IDH1,IDH2	2
Connective Tissue Disorders,Dermatological Diseases and Conditions,Developmental Disorder,Heredi	Autosomal recessive cutis laxa type 1A	1,02E-02			EFEMP2,FBLN5	2
Cell Death and Survival	Apoptosis of duct cancer cell lines	1,02E-02			SMO,TNFSF10	2
Developmental Disorder,Hereditary Disorder,Metabolic Disease,Organismal Injury and Abnormalities,	3-methylcrotonyl-CoA carboxylase 1 deficiency	1,02E-02			MCCC1,MCCC2	2
Cancer,Connective Tissue Disorders,Organismal Injury and Abnormalities,Skeletal and Muscular Disor	Chondrosarcoma in humerus	1,02E-02			IDH1,IDH2	2
Cell Cycle	Arrest in G2/M phase of pancreatic duct cells	1,02E-02			PPP2CA,PPP2CB	2
Cell Death and Survival,Connective Tissue Disorders,Organismal Injury and Abnormalities,Skeletal and	Apoptosis of articular chondrocytes	1,02E-02			SETDB1,TNFSF10	2
Cancer,Connective Tissue Disorders,Organismal Injury and Abnormalities,Skeletal and Muscular Disor	Chondrosarcoma in bone	1,02E-02			IDH1,IDH2	2
Endocrine System Disorders,Metabolic Disease,Organismal Injury and Abnormalities	Acquired secondary hypothyroidism	1,02E-02			THRA,THRB	2
Endocrine System Disorders,Metabolic Disease,Organismal Injury and Abnormalities	Acquired primary hypothyroidism	1,02E-02			THRA,THRB	2
Cell Cycle	Arrest in G1 phase of fibrosarcoma cell lines	1,02E-02			MAP3K5,RABL6	2
Endocrine System Disorders,Metabolic Disease,Organismal Injury and Abnormalities	Acquired tertiary hypothyroidism	1,02E-02			THRA,THRB	2
